# Evolution of pharmacologic specificity in the pregnane X receptor

**DOI:** 10.1186/1471-2148-8-103

**Published:** 2008-04-02

**Authors:** Sean Ekins, Erica J Reschly, Lee R Hagey, Matthew D Krasowski

**Affiliations:** 1 Collaborations in Chemistry, Inc., Jenkintown, PA, USA; 2 Department of Pharmaceutical Sciences, University of Maryland, Baltimore, MD, USA; 3 Department of Pharmacology, University of Medicine and Dentistry of New Jersey, Robert Wood Johnson Medical School, Piscataway, NJ, USA; 4 Department of Pathology, University of Pittsburgh, Pittsburgh, PA, USA; 5 Department of Medicine, University of California at San Diego, San Diego, CA, USA

## Abstract

**Background:**

The pregnane X receptor (PXR) shows the highest degree of cross-species sequence diversity of any of the vertebrate nuclear hormone receptors. In this study, we determined the pharmacophores for activation of human, mouse, rat, rabbit, chicken, and zebrafish PXRs, using a common set of sixteen ligands. In addition, we compared in detail the selectivity of human and zebrafish PXRs for steroidal compounds and xenobiotics. The ligand activation properties of the Western clawed frog (*Xenopus tropicalis*) PXR and that of a putative vitamin D receptor (VDR)/PXR cloned in this study from the chordate invertebrate sea squirt (*Ciona intestinalis*) were also investigated.

**Results:**

Using a common set of ligands, human, mouse, and rat PXRs share structurally similar pharmacophores consisting of hydrophobic features and widely spaced excluded volumes indicative of large binding pockets. Zebrafish PXR has the most sterically constrained pharmacophore of the PXRs analyzed, suggesting a smaller ligand-binding pocket than the other PXRs. Chicken PXR possesses a symmetrical pharmacophore with four hydrophobes, a hydrogen bond acceptor, as well as excluded volumes. Comparison of human and zebrafish PXRs for a wide range of possible activators revealed that zebrafish PXR is activated by a subset of human PXR agonists. The *Ciona *VDR/PXR showed low sequence identity to vertebrate VDRs and PXRs in the ligand-binding domain and was preferentially activated by planar xenobiotics including 6-formylindolo-[3,2-*b*]carbazole. Lastly, the Western clawed frog (*Xenopus tropicalis*) PXR was insensitive to vitamins and steroidal compounds and was activated only by benzoates.

**Conclusion:**

In contrast to other nuclear hormone receptors, PXRs show significant differences in ligand specificity across species. By pharmacophore analysis, certain PXRs share similar features such as human, mouse, and rat PXRs, suggesting overlap of function and perhaps common evolutionary forces. The Western clawed frog PXR, like that described for African clawed frog PXRs, has diverged considerably in ligand selectivity from fish, bird, and mammalian PXRs.

## Background

**Figure 1:**
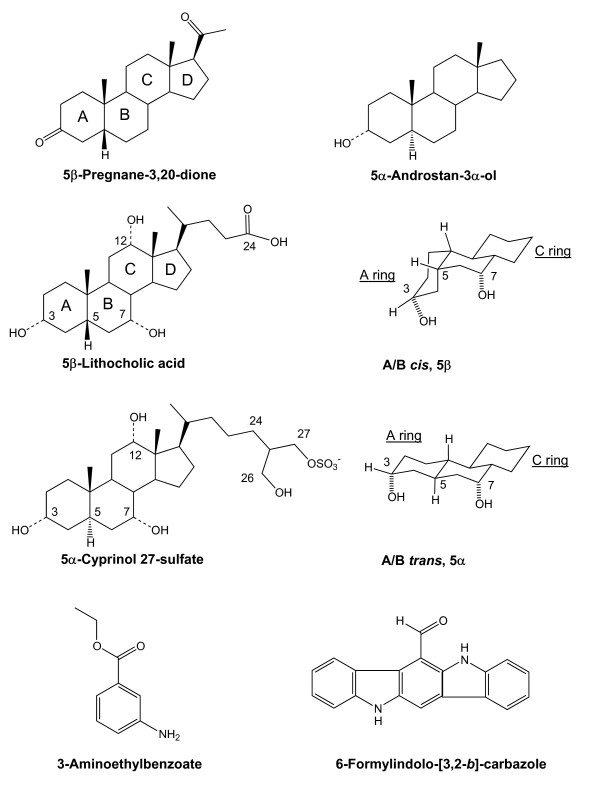
**Chemical structures of PXR activators**. Chemical structures of the PXR activators 5β-pregnane-3,20-dione, 5α-androstan-3α-ol, 5β-lithocholic acid, 5α-cyprinol 27-sulfate, 3-aminoethylbenzoate, and 6-formylindolo-[3,2-*b*]-carbozole. The key bond positions are numbered for the steroids and bile salts, and the lettering of the steroidal rings is indicated for pregnanedione and lithocholic acid. The structure to the right of lithocholic acid illustrates the most stable orientation of the A, B, and C steroid rings for 5β-bile salts (like lithocholic acid) with the A/B *cis *configuration (referring to the relative orientation of the hydrogen atom substituents on carbon atoms 5 and 10). The structure to the right of 5α-cyprinol sulfate shows the most stable orientation of 5α-bile salts (like 5α-cyprinol sulfate) that prefentially adopt the A/B *trans *configuration.

The pregnane X receptor (PXR; NR1I2; also known as steroid and xenobiotic receptor) is a member of the nuclear hormone receptor (NR) superfamily [1,2]. PXR functions as a ligand-activated transcription factor and regulates the metabolism, transport, and excretion of exogenous compounds, steroid hormones, vitamins, bile salts, and xenobiotics. A remarkably diverse array of compounds activate human PXR, although generally only at micromolar concentrations (less commonly at high nanomolar concentrations), consistent with a hypothesized function of PXR as a toxic compound sensor [3,4] (see Figure 1 for chemical structures of some PXR activators).

PXR genes have been cloned and functionally characterized from a variety of vertebrate species, including human, rhesus monkey, mouse, rat, rabbit, dog, pig, chicken, frog, and zebrafish [1,4-12]. Like other NRs, PXRs have a modular structure with two major domains: an N-terminal DNA-binding domain (DBD) and a larger C-terminal ligand-binding domain (LBD). The PXR LBD is unusually divergent across species, compared to other NRs, with only 50% sequence identity between mammalian and non-mammalian PXR sequences; other NRs tend to have corresponding sequence identities at least 10–20% higher [12,13]. Even the PXR DBD, which is more highly conserved across species than the LBD, shows more cross-species sequence diversity than other NRs [12-16]. A detailed phylogenetic analysis of the entire vertebrate NR superfamily demonstrated evidence of positive evolutionary selection for the LBD of PXRs [17].

In this study, we compare in detail the selectivity of human and zebrafish PXRs for steroid hormones and related compounds. We also compare human, mouse, rat, rabbit, chicken, frog, and zebrafish PXRs with a set of common compounds that activate most PXRs. These *in vitro *data are used to develop pharmacophore models to capture the essential structural and chemical features of activators of these PXRs (pharmacophore models summarize the key features important for biological activity). Commonly used features in pharmacophore models include hydrophobic, hydrogen bond acceptor, hydrogen bond donor, and excluded volumes (areas where atoms are not allowed, e.g., due to steric overlap with receptor amino acid residues).

We sought to probe the distant evolutionary history of PXR and the related vitamin D receptor (VDR; NR1I1) by studying an invertebrate NR1I-like receptor. The draft genome of the chordate invertebrate *Ciona intestinalis *(sea squirt; a urochordate) revealed a single gene [GenBank: BR000137] with close sequence similarity to the vertebrate VDRs, PXRs, and constitutive androstane receptors (CARs, NR1I3) [18,19] (see Additional file 1 for sequence alignment). NR1I-like genes were also detected in the genomes of the fruitfly (*Drosophila melanogaster*) and the nematode *Caenorhabditis elegans *[20], although these genes have yet to be functionally characterized. The draft genome of the purple sea urchin (*Strongylocentrotus purpuratus*) revealed several putative NR1H-like genes but no NR1I-like genes [21]. The early evolutionary history of the NR1I subfamily (VDR, PXR, CAR) in vertebrates is not completely clear, but one hypothesis is that a single ancestral 'VDR/PXR' gene duplicated, with the two genes then diverging into distinct VDRs and PXRs, both of which are currently found in both mammalian and non-mammalian species [22]. We follow the convention of referring to the non-mammalian PXR/CAR-like genes as PXRs [12], although it is not clear whether the function of the single gene in fishes and chicken is more similar to mammalian CAR or PXR [9,10]. The duplication of a single VDR/PXR gene into two different genes may have occurred during a complex series of gene duplications that are thought to have occurred in early vertebrate evolution, based on analysis of lamprey and hagfish genes [23]. Later in vertebrate evolution (probably early on in or before the evolution of mammals), a single PXR-like ancestral gene then duplicated with subsequent divergence into the separate PXR and CAR genes found in all mammals sequenced thus far [9]. For simplicity, the single *Ciona intestinalis *NR1I-like gene will be referred to as '*Ciona *VDR/PXR'. One advantage of studying *Ciona intestinalis*, in addition to the genome project data available, is that this animal is a member of Urochordata, a subphylum now thought to contain the closest extant relatives of modern vertebrates [24].

From the Ghost database of *Ciona intestinalis *Genomic and cDNA Resources [25], cDNA clone IDs ciem829d05 and cilv048e18 correspond to the *Ciona *VDR/PXR. Based on the expressed sequence tag counts, these cDNAs show highest expression in the larvae and juvenile life stages and lower expression in eggs, cleaving embryos, young adults, and mature adults. For adult animals, expression was seen in gonadal tissue and blood cells. Although invertebrates are not known to produce and utilize vitamin D pathways, we speculated that the *Ciona *VDR/PXR may bind ligands structurally similar to vitamin D, based on the subsequent evolutionary development and ligand preferences of vertebrate VDRs. Alternatively, the *Ciona *VDR/PXR may function more like vertebrate PXRs, and assist in protection from toxic levels of endogenous and/or exogenous compounds, in which case it might bind a diverse array of ligands. We therefore cloned and expressed the *Ciona *VDR/PXR to determine how similar this receptor is to vertebrate NR1I receptors in terms of activation by ligands.

## Results

### Selectivity of human PXR

We first assessed the ability of a diverse set of compounds to activate human PXR by determining detailed concentration-response curves for activation of human PXR for 25 androstane steroids (Table 1), 11 estrane steroids (Table 1), 29 pregnane steroids (Table 2), 50 bile salts (Additional file 2; some bile salts were previously published [15]), and 50 additional diverse compounds that included xenobiotics and vitamins (Table 3) (see Figure 1 for selected chemical structures of an androstane steroid, a pregnane steroid, two bile salts, and two additional compounds). These activation data further confirm the broad ligand specificity of human PXR, with most compounds only activating at micromolar concentrations.

**Table 1:** Activation of human and zebrafish PXRs by androstane and estrane steroids

Cmp. #	Compound	hPXR Activity	hPXR Efficacy	zfPXR Activity	zfPXR Efficacy	Toxicity
	**ANDROSTANES**					
AN1	5α-Androstan-3α,17β-diol	5.38	0.68	5.19	0.84	None
AN2	5α-Androstan-3,17-dione (androstanedione)	4.90	0.87	5.50	0.86	None
AN3	5α-Androstan-3α-ol (androstanol)	5.20	0.5	5.34	1.00	None
AN4	5α-Androstan-3α-ol-17-one (androsterone)	4.73	0.93	5.60	0.87	None
AN5	5α-Androstan-17β-ol-3-one (dihydrotestosterone)	4.94	0.39	5.21	0.59	None
AN6	5β-Androstan-3α-ol-17-one (etiocholanolone)	5.24	0.54	5.47	0.88	200
AN7	4-Androsten-3,17-dione (androstenedione)	4.69	0.59	5.44	0.14	None
AN8	4-Androsten-17β-ol-3-one (testosterone)	4.14	0.22	5.61	0.12	None
AN9	5-Androsten-3β-ol-17-one (DHEA)	4.49	0.52	4.89	0.35	None
AN10	5α-Androst-16-en-3α-ol (androstenol)	5.26	0.7	5.44	1.02	None
AN11	5β-Androstan-3α,11β-diol-17-one	4.72	0.51	4.52	1.04	None
AN12	5-Androsten-3β-sulfate-17-one (DHEA sulfate)	4.32	0.22	None		None
AN13	5β-Androstan-3α-ol-17-one (epiandrosterone)	5.31	0.7	5.02	0.43	None
AN14	5β-Androstan-3α-ol-11,17-dione	4.39	0.15	5.01	0.49	None
AN15	4-Androsten-17α-ol-3-one (epitestosterone)	4.17	0.9	None		None
AN16	4-Androsten-17α-glucosiduronate-3-one (epitestosterone glucuronide)	4.86	0.69	None		None
AN17	4-Androsten-17α-sulfate-3-one (epitestosterone sulfate)	5.47	0.67	None		None
AN18	5β-Androstan-3α-glucosiduronate-17-one (etiocholanolone glucuronide)	None		None		None
AN19	5α-Androstane	None		None		100
AN20	5α-Androstan-3β-ol	6.10	0.43	5.57	1.66	50
AN21	5α-Androst-16-en-3β-ol	5.32	1.01	5.48	2.11	50
AN22	5α-Androst-16-en-3-one	5.52	0.96	5.58	0.68	100
AN23	5β-Androstan-3α-ol	5.85	1.12	5.59	0.33	None
AN24	Androst-4,16-dien-3-one	5.15	0.64	5.96	0.17	100
AN25	Androst-5,16-dien-3β-ol	None		5.60	1.50	100
						
	**ESTRANES**					
						
ES1	1,3,5(10)-Estratrien-3,17β-diol (estradiol)	4.80	0.34	None		200
ES2	1,3,5(10)-Estratrien-3-ol-17-one (estrone)	4.42	0.47	None		200
ES3	1,3,5(10)-Estratrien-3,16α,17β-triol (estriol)	None		None		200
ES4	1,3,5(10)-Estratrien-3,16α-diol-17-one (16α-hydroxyestrone)	5.60	0.42	5.70	0.17	None
ES5	1,3,5(10)-Estratrien-3-ol-4-methoxy-17-one (4-methoxyestrone)	5.40	0.93	5.62	0.19	None
ES6	1,3,5(10)-Estratrien-3,15α,16α,17β-tetrol (estetrol)	5.67	0.29	None		200
ES7	1,3,5(10)-Estratrien-2,3-diol-17-one (2-hydroxyestrone)	5.44	0.93	5.74	0.19	None
ES8	1,3,5(10)-Estratrien-17-one-3-sulfate (estrone sulfate)	5.47	0.43	None		None
ES9	1,3,5(10)-Estratrien-17β-ol-3-glucosiduronate (estradiol glucuronide)	None		None		None
ES10	1,3,5(10)-Estratrien-17β-ol-3-sulfate (estradiol sulfate)	6.05	0.6	None		200
ES11	1,3,5(10)-Estratrien-17α-ethinyl-3,17β-diol (ethinyl estradiol)	5.72	0.68	None		200

Activities are in -log(EC_50_), with EC_50 _in molar units for the activation of human or zebrafish PXR. Efficacy is relative to 10 μM rifampicin (human PXR) or 20 μM 5α-androstan-3α-ol (zebrafish PXR) which are assigned an efficacy of 1.0. Toxicity is the lowest concentration in micromolar that produced significant toxicity in the HepG2 cells.

**Table 2:** Activation of human and zebrafish PXRs by pregnane steroids and related compounds

Cmp. #	Compound	hPXR Activity	hPXR Efficacy	zfPXR Activity	zfPXR Efficacy	Toxicity
PR1	5β-Pregnan-3α,20α-diol (5β-pregnanediol)	5.29	0.34	None		100
PR2	5β-Pregnan-3,20-dione (5β-pregnanedione)	5.59	0.97	6.08	0.85	None
PR3	4-Pregnen-11β,21-diol-3,20-dione (corticosterone)	5.00	0.54	None		None
PR4	4-Pregnen-17,21-diol-3,20-dione (cortexolone)	4.64	0.49	None		None
PR5	4-Pregnen-11β,21-diol-3,18,20-trione (aldosterone)	4.26	0.21	None		None
PR6	4-Pregnen-17,21-diol-3,11,20-trione (cortisone)	4.16	0.28	None		200
PR7	4-Pregnen-3,20-dione (progesterone)	4.83	0.57	None		200
PR8	4-Pregnen-17-ol-3,20-dione	4.75	0.7	None		None
PR9	4-Pregnen-21-ol-3,20-dione (cortexone)	5.61	0.3	None		200
PR10	4-Pregnen-3β,17,21-triol-3,20-dione (cortisol)	4.32	0.66	None		None
PR11	5-Pregnen-3β,17-diol-20-one	4.47	0.36	None		None
PR12	5-Pregnen-3β-diol-20-one (pregnenolone)	5.64	1.26	6.32	2.05	None
PR13	5-Pregnen-16α-cyano-3β-ol-20-one	None		None		None
PR14	5α-Pregnan-3α-ol-20-one (allopregnanolone)	5.38	0.46	5.40	0.30	None
PR15	5α-Pregnan-3α,20α-diol (allopregnanediol)	4.28	0.16	4.58	0.29	None
PR16	5β-Pregnan-3α,20α-diol-3-glucosiduronate (pregnanediol glucuconide)	4.26	0.17	4.78	1.42	None
PR17	5β-Pregnan-3α,11β,17,20α-21-pentol (cortol)	4.33	0.83	3.95	0.98	200
PR18	5β-Pregnan-3α,17,20α-21-tetrol-11-one (cortolone)	4.35	0.72	4.34	0.25	200
PR19	5β-Pregnan-3α,17,21-triol-11,20-dione	4.28	0.8	4.43	0.33	None
PR20	5α-Pregnan-3α,11β,21-triol-20-one	4.90	0.26	None		None
PR21	4-Pregnen-17α,20β-diol-3,20-dione	None		None		200
PR22	4-Pregnen-20β-ol-3,20-dione-17α-sulfate	5.73	0.66	None		None
PR23	5β-Pregnan-3α,20β-diol	5.42	0.49	5.95	0.25	None
PR24	5β-Pregnan-3α,11β,17,21-tetrol-20-one	4.33	0.73	3.94	0.11	None
PR25	5β-Pregnan-3α-ol-20-one	4.98	0.55	6.64	1.22	100
PR26	5-Pregnen-20-one-3β-sulfate	None		4.95	0.23	None
PR27	4-Estren-17α-ethynyl-18-homo-17β-ol-3-one (levonorgestrel)	5.37	0.35	6.00	0.86	None
PR28	4-Estren-17α-ethynyl-17β-ol-3-one (norethindrone)	4.59	0.32	5.85	0.40	None
PR29	1,4-Pregnadien-9α-fluoro-16α-methyl-11β,17,21-triol-3,20-dione (dexamethasone)	4.39	0.83	None		None

Activities are in -log(EC_50_), with EC_50 _in molar units for the activation of human or zebrafish PXR. Efficacy is relative to 10 μM rifampicin (human PXR) or 20 μM 5α-androstan-3α-ol (zebrafish PXR) which are assigned an efficacy of 1.0. Toxicity is the lowest concentration in micromolar that produced significant toxicity in the HepG2 cells.

**Table 3:** Activation of human and zebrafish PXRs by xenobiotics and vitamins

Cmp. #	Compound	hPXR Activity	hPXR Efficacy	zfPXR Activity	zfPXR Efficacy	Toxicity
MI1	Acetaminophen	None		None		None
MI2	3-Aminobenzoic acid	None		None		None
MI3	Benzo [a]pyren	4.75	0.55	4.00	0.06	100
MI4	*n*-Butyl-4-aminobenzoate	4.88	1.35	4.86	0.69	100
MI5	Butylbenzoate	None		None		None
MI6	Caffeine	None		None		None
MI7	Carbamazepine	4.20	0.37	None		200
MI8	Carbamazepine epoxide	4.09	0.57	None		200
MI9	β-Carotene	5.46	0.67	None		100
MI10	Chlorpyrifos	4.59	2.05	5.44	0.88	None
MI11	Chlorzoxazone	None		None		500
MI12	Cyclosporine	None		None		20
MI13	Ecdysone	None		None		None
MI14	Ethyl-2-aminobenzoate	None		None		None
MI15	Flurbiprofen	4.10	1.59	4.10	0.53	None
MI16	Folic acid	None		None		None
MI17	Guggulsterone	None		None		None
MI18	GW3965	None		None		10
MI19	Hyperforin	7.22	1.29	None		50
MI20	Mevastatin	5.23	0.51	None		15
MI21	Mycophenolic acid	None		None		None
MI22	Nifedipine	5.33	0.41	4.91	0.99	50
MI23	Oxcarbazepine	4.74	0.35	~4.70	~0.30	200
MI24	Paclitaxel	4.92	0.13	None		100
MI25	Phenobarbital	3.43	1.19	3.49	0.10	None
MI26	Phenytoin	4.26	0.52	None		200
MI27	*n*-Propyl-4-hydroxybenzoate	4.51	0.32	4.28	0.31	100
MI28	Provitamin D_3_	None		None		10
MI29	Provitamin D_2_	None		None		20
MI30	Reserpine	4.91	0.72	None		50
MI31	Retinol	5.80	0.20	None		50
MI32	Rifampicin	7.00	1.00	None		200
MI33	SR12813	6.41	0.90	None		10
MI34	TCDD	7.17	1.78	6.32	6.17	10
MI35	TCPOBOP	5.25	0.66	None		200
MI36	T-0901317	7.66	1.24	None		100
MI37	α-Tocopherol	~4.30	~0.25	None		100
MI38	β-Tocopherol	4.85	0.33	None		100
MI39	δ-Tocopherol	5.14	0.64	None		100
MI40	γ-Tocopherol	None		None		100
MI41	1α,25-Dihydroxyvitamin D_3_	None		None		50
MI42	1α-Hydroxyvitamin D_2_	None		None		50
MI43	1α-Hydroxyvitamin D_3_	None		None		50
MI44	Vitamin K_1_	4.99	0.13	None		100
MI45	Vitamin K_2_	5.04	0.80	None		100
MI46	Vitamin K_3_	~4.30	~0.15	None		100

Activities are in -log(EC_50_), with EC_50 _in molar units for the activation of human or zebrafish PXR. Efficacy is relative to 10 μM rifampicin (human PXR) or 20 μM 5α-androstan-3α-ol (zebrafish PXR) which are assigned an efficacy of 1.0. Toxicity is the lowest concentration in micromolar that produced significant toxicity in the HepG2 cells.

### Comparison of human and zebrafish PXRs

**Figure 2:**
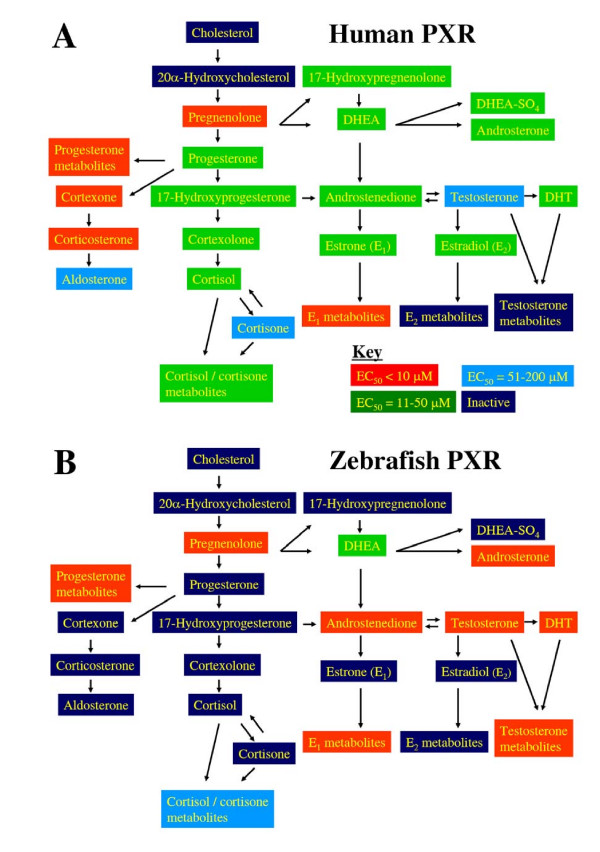
**PXR activation and steroid pathways**. Steroid pathways typical of vertebrates are indicated. (A) Human PXR is activated by a large number of steroid hormones, although typically at micromolar concentrations. The coloring indicates at which concentrations the various steroids activate human PXR (see key in bottom right of panel). (B) Zebrafish PXR is activated by a smaller number of steroid hormones than human PXR, although there is much overlap between the selectivity of the two PXRs. Zebrafish PXR tends to be more sensitive to steroid hormone activation, at least for the functional assay used in this study. The coloring indicates at which concentrations the various steroids activate zebrafish PXR using the same key as in (A). Abbreviations: dehydroepiandrosterone, DHEA; DHEA sulfate; DHEA SO_4_; dihydrotesterone, DHT.

In two prior studies that compared PXRs from different species, human and zebrafish PXRs were found to share some activating ligands, including pregnanes, androstanes, and a few xenobiotics such as nifedipine and phenobarbital [12,15]. Activation of zebrafish PXR by the much larger set of 165 compounds tested on human PXR was determined in this study, and these two species showed considerable overlap in their ligand specificity (Tables 1, 2, 3, Additional file 2). Human PXR has very broad specificity for steroid hormones and their synthetic intermediates (Figure 2A) albeit mostly at micromolar concentrations likely to exceed typical physiologic concentrations [2,8].

Zebrafish PXR was activated by far fewer steroid compounds which were essentially a subset of those that activate human PXR (Figure 2B). For both human and zebrafish PXRs, pregnane steroids showed the highest activity (Figure 2, Table 2). Human and zebrafish PXRs showed more differences in regard to bile salt activators with zebrafish PXR being activated by very few of the bile salts tested (Additional file 2). In terms of the evolution of bile salts, human PXR is activated by both evolutionary 'early' bile salts [26-28] (e.g., 27-carbon bile alcohol sulfates such as 5α-cholestan-3α,7α,12α,26,27-pentol [cyprinol] 27-sulfate) and 'recent' bile salts (e.g., cholic acid) (Additional files 2 and 3). Zebrafish PXR is activated only by early bile salts, including 5α-cyprinol sulfate and 5β-scymnol (5β-cholestan-3α,7α,12α,24,26,27-hexol) 27-sulfate (Additional files 2 and 3). The results are consistent with crystallographic studies of human PXR that show a large, flexible ligand-binding pocket [29-34]. This pocket can accommodate bile salts of both 5α (A/B *trans*) and 5β (A/B *cis*) orientation (Figure 1), as well as those with differing side-chain lengths and conjugation. This is in contrast to studies of farnesoid X receptors (FXRs; NR1H4) and VDRs, two other NRs that are activated by bile acids [35-38]. In particular, FXRs are antagonized by 5α-bile alcohol sulfates [39] while VDRs are essentially only activated by the smallest bile salt, lithocholic acid (5β-cholan-3α-ol-24-oic acid), and its metabolites [38,40,41].

### Pharmacophore models for six PXRs

**Figure 3:**
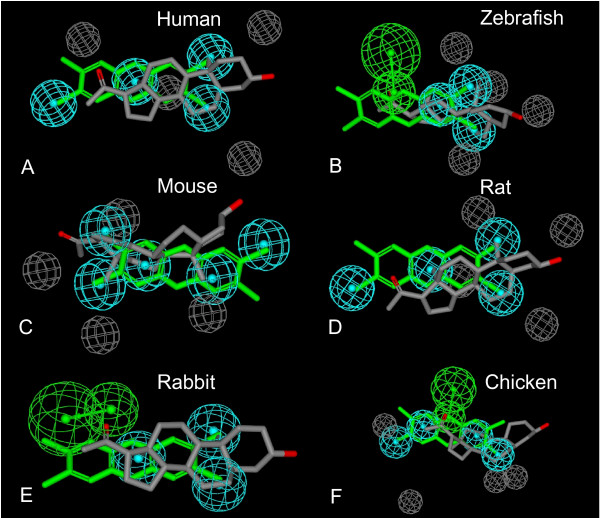
**Pharmacophore models of PXR activators**. Pharmacophore models of PXR activators of (A) human PXR, (B) zebrafish PXR, (C) mouse PXR, (D) rat PXR, (E) rabbit PXR, and (F) chicken PXR. The pharmacophores were generated from the same 16 molecules using Catalyst. The molecules mapped to each pharmacophore are TCDD (green) and 5β-pregnane-3,20-dione (grey). It should be noted that TCDD is inactive in rabbit PXR and only maps to the hydrophobic features. The pharmacophore features are hydrophobic (cyan), hydrogen bond acceptor and vector (green), and excluded volume (grey).

In a comparative study, we determined concentration-response curves for a common set of 16 compounds (steroids, bile salts, xenobiotics) in a set of PXRs from six species (human, zebrafish, mouse, rat, rabbit, and chicken; Tables 1, 2, 3, 4, Additional file 2). The pharmacophores generated are shown mapped to two of the generally more active ligands, 2,3,7,8-tetrachlorodibenzo-*p*-dioxin (TCDD) and 5β-pregnane-3,20-dione (Figure 3). Human, rat, and mouse PXRs showed very similar pharmacophores with 4–5 hydrophobic features and multiple excluded volumes (Figure 3A,C,D). The pharmacophores for these three PXRs all suggest generally large ligand-binding pockets with differences only in positions of the features. It is interesting that compared with previous pharmacophores for human PXR [42-44] which contained 4–5 hydrophobic features and at least 1–2 hydrogen bonding moieties, there are no hydrogen bonding features in the current human PXR pharmacophore. This could be due to the molecules used in the current training set being mostly bile salts and having active and inactive compounds with similar features. As the Catalyst pharmacophore generation method looks for differences between the extremes of activity to describe the features contributing to the pharmacophore, this may represent a limitation of the method. While a single universal pharmacophore for human PXR (and perhaps PXRs from other species) may be impossible due to the size and flexibility of the binding site, it is likely in the current study that the 16 selected molecules may just be a sub-section of the binding pocket. For example, this may be where steroidal compounds fit [33] as modelled previously with a pharmacophore [45]. Therefore, the pharmacophores serve as a novel qualitative method for analysis of PXR ligand specificity across the species.

**Table 4:** Activation of mouse, rat, rabbit, and chicken PXRs

Cmp #	Compound	**Mouse PXR Activity (efficacy, ε, in parentheses)**	**Rat PXR Activity (efficacy, ε, in parentheses)**	**Rabbit PXR Activity (efficacy, ε, in parentheses)**	**Chicken PXR Activity (efficacy, ε, in parentheses)**
**BI004**	**Murideoxycholic acid**	5.09 (ε = 0.76)	4.80 (ε = 0.22)	4.52 (ε = 1.86)	No effect
**BI005**	**Chenodeoxycholic acid**	No effect	No effect	4.70 (ε = 0.42)	4.70 (ε = 0.36)
**BI008**	**Deoxycholic acid**	No effect	5.00 (ε = 0.32)	4.44 (ε = 0.37)	No effect
**BI011**	**Lithocholic acid**	4.86 (ε = 0.48)	4.78 (ε = 0.42)	4.80 (ε = 0.70)	5.09 (ε = 0.17)
**BI020**	**Cholic acid**	No effect	4.82 (ε = 0.42)	4.02 (ε = 0.67)	4.47 (ε = 0.36)
**BI023**	**5β-Cholestan-3α,7α,12α-triol**	5.85 (ε = 1.23)	5.65 (ε = 0.72)	5.41 (ε = 0.37)	5.89 (ε = 0.27)
**BI034**	**5β-Scymnol sulfate**	4.44 (ε = 0.85)	4.40 (ε = 0.85)	4.09 (ε = 1.93)	4.37 (ε = 0.88)
**BI036**	**5α-Cyprinol sulfate**	4.78 (ε = 0.29)	4.50 (ε = 0.28)	4.09 (ε = 0.43)	4.51 (ε = 0.61)
**BI038**	**3α,7α,12αtTrihydroxy-5β-cholestan-27-oic acid, taurine conjugated**	No effect	No effect	No effect	No effect
**BI046**	**Tauro-β-muricholic acid**	No effect	No effect	No effect	No effect
**BI047**	**7α-Hydroxycholesterol**	No effect	No effect	No effect	No effect
**PR2**	**5β-Pregnane-3,20-dione**	5.36 (ε = 0.84)	5.24 (ε = 1.01)	4.90 (ε = 1.0)	5.59 (ε = 0.81)
**MI3**	**Benzo [a]pyren**	4.86 (ε = 0.94)	4.85 (ε = 0.45)	No effect	4.40 (ε = 0.50)
**MI4**	***n*-Butyl-*p*-aminobenzoate**	No effect	< 4	< 4	> 100
**MI22**	**Nifedipine**	4.64 (ε = 0.51)	5.26 (ε = 0.68)	4.61 (ε = 0.29)	6.14 (ε = 1.00)
**MI34**	**TCDD (2,3,7,8-tetrachlorodienzo-*p*-dioxin)**	7.00 (ε = 1.60)	6.70 (ε = 0.83)	No effect	7.04 (ε = 0.06)
AN1	5α-Androstan-3α-,17β-diol				5.07 (ε = 2.28)
AN3	5α-Androstan-3α-ol				5.38 (ε = 0.85)
AN21	5α-Androst-16-en-3β-ol				5.14 (ε = 2.99)
AN22	5α-Androst-16-en-3-one				4.99 (ε = 0.77)
BI031	Allocholic acid				No effect
BI006	Glycochenodeoxycholic acid				4.60 (ε = 0.41)
BI007	Taurochenodeoxycholic acid				No effect
BI009	Glycodeoxycholic acid			4.81 (ε = 0.40)	
BI010	Taurodeoxycholic acid			4.84 (ε = 0.15)	
BI017	ω-Muricholic acid	No effect	No effect		
BI018	α-Muricholic acid	4.59 (ε = 2.63)	3.95 (ε = 1.31)		
BI019	β-Muricholic acid	No effect	No effect		
BI021	Glycocholic acid	No effect	No effect		No effect
BI022	Taurocholic acid	4.07 (ε = 0.93)	No effect		No effect
BI042	7-Ketodeoxycholic acid			4.31 (ε = 1.55)	
PR13	Pregenolone 16α-carbonitrile	6.41 (ε = 1.0)	6.20 (ε = 1.0)		

Activities are in -log(EC_50_), with EC_50 _in molar units for the activation of mouse, rat, rabbit, or chicken PXRs. Efficacy is relative to 20 μM pregnenolone 16α-carbonitrile (mouse and rat PXRs), 50 μM 5α-pregnan-3,20-dione (rabbit PXR), or 20 μM nifedipine (chicken PXR) which are assigned an efficacy of 1.0. The training set consists of the 16 compounds highlighted in **bold **font.

Zebrafish PXR showed the most constrained pharmacophore based on the 16 ligands, suggesting a small binding pocket compared with the other PXRs, consisting of 3 hydrophobes, 1 hydrogen bond acceptor, and excluded volumes (Figure 3B). Rabbit PXR had a similar pharmacophore model to zebrafish PXR but no excluded volumes as in the former (Figure 3E). Chicken PXR had a pharmacophore qualitatively different from the other PXRs, with the model indicating a symmetrical array of features that contribute to activity (Figure 3F); it is perhaps noteworthy that this PXR has a smaller 'insert' sequence between helices 1 and 3 of the LBD than that of human, mouse, rat, and rabbit PXRs [9,12]. The pharmacophore models for both chicken and zebrafish PXRs also show a hydrogen bond acceptor not found in the models for PXRs from other species (Figure 3); this hydrogen bonding interaction may contribute to the relatively high activity of TCDD in chicken and zebrafish PXRs. Pharmacophore statistical summaries are presented in Additional file 4.

### Unusual pharmacology of *Xenopus *frog PXRs

Whereas other vertebrates such as human, mouse, rat, chicken, and zebrafish have a single PXR gene in their respective genomes, two PXRs have been identified in the African clawed frog (*Xenopus laevis*) [7,46]. This is likely a consequence of the tetraploidy of the *X. laevis *genome [47]. The phylogeny confirms that these two genes are *bone fide *orthlogs to mammalian PXR; however their pharmacology and tissue expression pattern is markedly different [7,46,48,49]. *Xenopus laevis *PXRs are alternatively termed 'benzoate X receptors' (BXRs) due to their activation by endogenous benzoates (such as 3-aminoethylbenzoate; Figure 1) that play a role in frog development [7]. Similar benzoates have yet to be characterized in other animals, suggesting that these may be unique to amphibians. In addition to PXRs, other gene families show divergence in *Xenopus laevis *relative to other vertebrates. Per-ARNT-Sim (PAS) proteins such as the aryl hydrocarbon receptor (AHR) nuclear translocator are an example [50].

Our search of the sequenced genome of the related Western clawed frog (*Xenopus tropicalis*; an animal with a diploid genome) revealed only a single PXR gene. Cloning of the LBD of this PXR from adult female ovary and expression in a GAL4-LBD chimeric construct allowed for determination of ligand specificity. Similar to studies of the *Xenopus laevis *PXRs, the *Xenopus tropicalis *PXR was insensitive to steroids, vitamins, and xenobiotics that activate mammalian or chicken PXRs, but was activated by two benzoates described as activators of the *Xenopus laevis *PXRα (Additional File 5) [7,49].

### Properties of the *Ciona intestinalis *VDR/PXR

**Figure 4:**
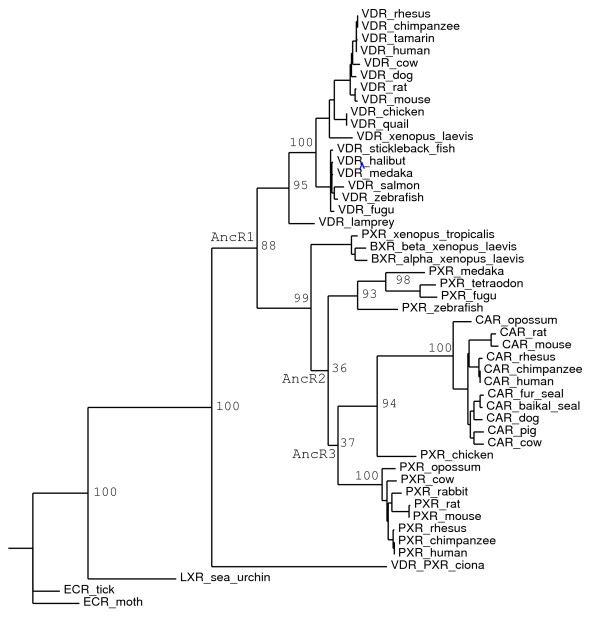
**Maximum likelihood phylogeny of VDRs, PXRs, and CARs**. Maximum likelihood phylogeny of 49 amino acid sequences of VDRs, PXRs, and CARs (see Methods for details of analysis). Numbered branch labels indicate bootstrap percentages. Node labels 'AncR1', 'AncR2', and 'AncR3' indicate ancestral nodes that were reconstructed (see Additional files 7 and 8).

Sequencing of the *Ciona intestinalis *genome revealed a single gene with similarity to vertebrate NR1I genes VDR, PXR, and CAR [18,19]. We previously reported a preliminary analysis of the *Ciona *VDR/PXR [51] and now present more detailed data. While the DBD of the *Ciona *VDR/PXR can be easily aligned to the corresponding sequence of vertebrate VDRs, PXRs, and CARs, alignment of the LBD is difficult in some regions (Additional file 1). As summarized in Table 5, the LBD of *Ciona *VDR/PXR has low sequence identity to vertebrate VDRs, PXRs, and CARs (17.1%–26.8%). In the DBD, the *Ciona *VDR/PXR has the highest sequence identity to sea lamprey and zebrafish VDRs (Table 5). The phylogeny of the *Ciona *VDR/PXR, as inferred by maximum likelihood analysis, does not clearly group this receptor with either VDRs or PXRs (Figure 4). This likely indicates that more sequences are needed, especially additional NR1I receptors (if present) in basal vertebrates (such as Agnatha) and chordate invertebrates. The low sequence identity between the *Ciona *VDR/PXR and vertebrate VDRs, PXRs, and CARs may be a result of rapid evolution, which has been detected in some gene families (including developmental regulators) in *Ciona intestinalis *and other tunicates [18,19,52,53].

**Table 5:** Sequence Identities of the *Ciona *VDR/PXR to Other Nuclear Hormone Receptors

Receptor	% Identity to *Ciona *VDR/PXR in DBD	% Identity to *Ciona *VDR/PXR in LBD
Human PXR	61.8	22.5
Mouse PXR	60.3	21.5
Chicken PXR	63.2	23.7
*Xenopus laevis *PXRα	64.7	20.3
Fugu PXR	67.6	19.8
		
Human VDR	67.6	17.1
Zebrafish VDR	**70.6**	21.8
Sea lamprey VDR	**73.5**	20.8
		
Human CAR	60.3	26.8
Mouse CAR	55.9	23.2
		
Human FXR	54.4	23.1
Zebrafish FXR	55.9	24.0
*Ciona *FXR	55.2	21.9
		
Human LXRa	54.4	21.9
Zebrafish LXR	54.4	19.8
*Ciona *LXR	52.9	19.3

The LBD of *Ciona *VDR/PXR was cloned from cDNA fragments generously provided by Professors Yuji Kohara and Norituki Satoh, and then inserted into the PM2-GAL4 plasmid to create an LBD/GAL4 chimeric receptor. Unlike similar constructs derived from the vertebrate VDRs, this *Ciona *VDR/PXR was not activated by any vitamin D derivatives, vertebrate bile salts, or steroid hormones (Additional file 5). Screening of a 76-compound nuclear hormone receptor ligand library (BIOMOL International, Plymouth Meeting, PA, USA) revealed that 6-formylindolo-[3,2-*b*]carbazole activated *Ciona *VDR/PXR in the low micromolar range (Additional file 5). 6-Formylindolo-[3,2-*b*]carbazole (20 μM) was chosen as the reference maximal activator of *Ciona *VDR/PXR. Screening of an additional 90 compounds comprising steroid hormones, vitamins (other than vitamin D), benzoates, and xenobiotics revealed that carbamazepine and *n*-butyl-*p*-aminobenzoate also activated *Ciona *VDR/PXR in the micromolar range (Additional file 5). Interestingly, 6-formylindolo-[3,2-*b*]carbazole, carbamazepine, and *n*-butyl-*p*-aminobenzoate are all planar molecules.

The Catalyst pharmacophore approach can also be used to generate common feature (HIPHOP) alignments [54] of the three molecules that active *Ciona *VDR/PXR. In this case the pharmacophore consisted of 1 hydrogen bond acceptor and 2 hydrophobic areas (Additional file 6). This pharmacophore is generally quite different compared with the models for other PXRs described above and in many ways reflects the very narrow substrate selectivity compared with the other six species.

### Phylogenetic analysis and ancestral reconstruction of NR1I receptors

Compared to other vertebrate NR subfamilies, the evolutionary history of the NR1I subfamily is difficult to reconstruct due to a high degree of functional and sequence divergence [10,12,22]. Some studies speculate that an ancestral gene duplicated early in vertebrate evolution (or possibly even prior to evolution of vertebrates), with subsequent divergence to become separate PXR and VDR genes [9,10,12,15,17,20,22,51]. Later in vertebrate evolution, a single PXR gene duplicated, with subsequent divergence to form separate PXR and CAR genes [10]. Throughout this manuscript, we follow the convention of designating the non-mammalian PXR/CAR-like genes as PXRs [12], although it is not certain that the ancestral PXR/CAR-like gene is actually the same gene now called PXR in mammals [9,10,20].

Using 49 amino acid sequences of extant VDRs, PXRs, and CARs, we inferred phylogeny by maximum likelihood (Figure 4). Several clusters are clearly evident and supported by bootstrap analysis in the phylogeny presented in Figure 4: vertebrate VDRs, mammalian CARs, and mammalian PXRs. The major difficulty is the placement of the frog PXRs, which are quite different from other PXRs in function, tissue expression, and sequence [7,46,48,49]. The chicken PXR clusters with the CARs in Figure 4; however, by many measures, chicken PXR is equally related to mammalian CARs and PXRs [9,10,12].

We also utilized maximum likelihood to infer the amino acid sequence of three 'ancestral' receptors, indicated as nodes in Figure 4 labelled as 'AncR1', 'AncR2', and 'AncR3' (Additional file 7). AncR1 represents the ancestral single receptor gene prior to duplication and subsequent divergence to VDRs and PXRs. AncR2 represents the PXR gene ancestral to the split between fish PXRs and mammalian CARs/PXRs. AncR3 represents the ancestral single receptor gene prior to duplication and subsequent divergence to mammalian PXRs and chicken PXR/mammalian CARs. It should be pointed out that ancestral reconstruction based on receptors that are markedly divergent in sequence, particularly when there are insertions or deletions of receptors relative to one another, is subject to significant uncertainly and should be interpreted cautiously. The inter-helical regions of the LBD are particularly difficult to predict. For the LBD, the percentage of amino acid residues with posterior probability greater than 0.7 is only 56.4%, 80.4%, and 65.0% in AncR1, AncR2, and AncR3, respectively (Additional file 7). These overall posterior probabilities are significantly lower than reconstruction of ancestral sex and mineralocortoid NRs (in the NR3 family) [55-57], where the cross-species sequence divergences are much less than for the NR1I subfamily of receptors. These uncertainties make homology modelling (or even functional expression) of the LBDs of reconstructed NR1I ancestral sequences unreliable. Therefore, we focused on cross-sequence comparisons of amino acid residues identified as interacting with ligands in crystal structures of human VDR [58-60], rat VDR [61,62], zebrafish VDR [63], human PXR [29-34], human CAR [64,65], and mouse CAR [66]. In this subset of 'ligand-binding residues', the percentage of amino acid residues with posterior probability greater than 0.7 is 56.8%, 90.2%, and 80.4% in AncR1, AncR2, and AncR3, respectively (Additional file 8); each of these values is higher than for the overall LBD sequence indicated above.

**Figure 5:**
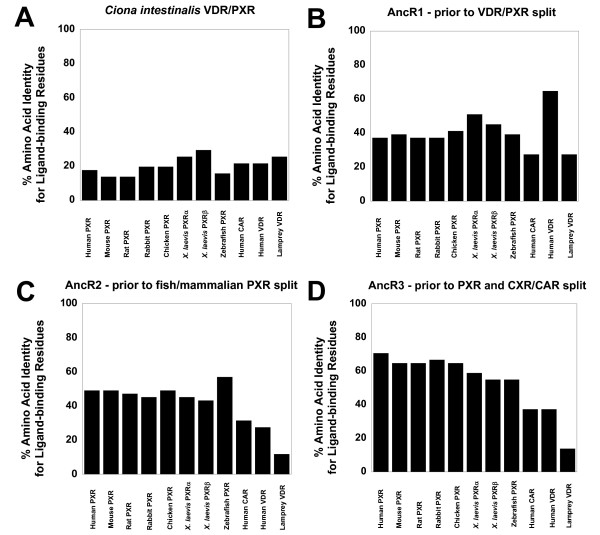
**Conservation of ligand-binding residues**. From published X-ray crystallographic structures of human VDR, rat VDR, zebrafish VDR, human PXR, human CAR, and mouse CAR (see Methods for references), amino acid residues that interact with ligands ('ligand-binding residues') were identified. At these amino acid residue positions, the sequences of *Ciona intestinalis *VDR/PXR, AncR1, AncR2, and AncR3 were compared with the corresponding sequence for human PXR, mouse PXR, rat PXR, rabbit PXR, chicken PXR, *Xenopus laevis *PXRα, *Xenopus laevis *PXRβ, zebrafish PXR, human CAR, human VDR, and sea lamprey VDR. The ordinate represents the percent identity of *Ciona intestinalis *VDR/PXR, AncR1, AncR2, and AncR3 for the corresponding sequences of PXRs, VDRs, or CAR at these ligand-binding residue positions.

At the amino acid residue positions identified as ligand-binding residues, we compared *Ciona *VDR/PXR, AncR1, AncR2, and AncR3 to mammalian PXRs (human, mouse, rat, rabbit), chicken PXR, *Xenopus laevis *PXRα and PXRβ, zebrafish PXR, human CAR, human VDR, and sea lamprey VDR (Figure 5). As with overall sequence comparisons in the LBD (Table 5), sequence identities at ligand-binding residues for *Ciona *VDR/PXR compared to the other receptors were overall low (< 25%). Interestingly, AncR1 showed the highest sequence identity to human VDR (64.7%); all other sequences were less than 51% identical to AncR1 (Figure 5 and Additional file 8). This would be consistent with VDR being the ancestral NR1I receptor [51]. The differences between *Ciona *VDR/PXR and AncR1 at the ligand-binding residue positions may be explained by rapid evolution of the *Ciona *gene, as discussed above. AncR2 had the highest sequence identity at ligand-binding residues to zebrafish PXR (56.9%), compared to ~ 45–50% for other PXRs and only 31.4% to human CAR. AncR3 had highest sequence identity at ligand-binding residues to mammalian PXRs (66.7% to 70.6%) compared to only 37.3% to human CAR (Figure 5 and Additional file 8). The results for AncR2 and AncR3 both suggest that CAR has diverged the most from the ancestral sequence at ligand-binding residues and would be consistent with PXR being the ancestral gene.

### Intrinsic disorder analysis

A key factor in protein interactions with ligands or other proteins is presence of intrinsic structural disorder [67,68]. To assess whether disorder may account for pharmacological differences between the PXRs from different species, intrinsic disorder of the amino acid residues were predicted using the PONDR VL3H algorithm [68] and summarized by the percentage of residues with probability of disorder greater than 50%. Disorder probabilities were analyzed by domain (DBD or LBD) or total protein sequence (Additional files 9 and 10). Rabbit PXR was shown to possess lower predicted intrinsic disorder in the LBD compared with human, mouse, rat, chicken, and zebrafish PXRs. The African clawed frog PXRα (BXRα) had the lowest predicted intrinsic disorder in the LBD of any PXR (Additional files 9 and 10); as discussed above, this receptor has very restricted ligand specificity, essentially responding only to benzoates (and their close structural analogs) shown to be important in early frog development [7]. In terms of intrinsic disorder, *Ciona *VDR/PXR was closer to PXRs than to VDRs in the LBD (Additional files 9 and 10). The chicken PXR was distinct from the other PXRs in terms of low intrinsic disorder in the DBD; in this regard, chicken PXR is much more similar to CARs (Additional files 9 and 10). This is consistent with the hypothesis that an ancestral gene very similar to chicken PXR duplicated, with the two genes ultimately diverging into separate CAR and PXR genes (chicken PXR has about equal sequence similarity to mammalian CARs and PXRs) [9,10,12]. In the DBD, chicken PXR may have structural features more similar to mammalian CARs than PXRs. The results are consistent with differences in structural disorder possibly contributing to differences in pharmacologic specificity.

## Discussion

PXRs show unusually low sequence conservation in the LBD across vertebrate species relative to other NRs [12,13,17]. Several groups have hypothesized that cross-species differences in the presence and utilization of endogenous and/or exogenous ligands have provided the evolutionary force for this divergence [8,15,69-71]. In this study, we have generated considerable new *in vitro *data that has enabled us to determine pharmacophore models for activation of six PXRs (human, mouse, rat, rabbit, chicken, and zebrafish) using a common set of 16 compounds. The pharmacophore models of human, mouse, and rat PXRs are quite similar overall, while the pharmacophore models for zebrafish and chicken PXRs are significantly different compared with those for the mammalian PXRs. The *in vitro *and modelling data support a smaller ligand-binding pocket for zebrafish PXR. Our data for the Western clawed frog PXR show that this receptor, similar to African clawed frog PXRs [7,49], may be sensitive only to benzoates and close analogs.

We also report the first characterization of the *Ciona intestinalis *VDR/PXR. Sequencing of the *C. intestinalis *genome reveals only a single NR1I-like gene, along with two NR1H-like genes [19]. The *Ciona *'VDR/PXR' has substantially less sequence identity in the LBD to either VDR or PXR than in the DBD, and the receptor was not activated by any of the steroids, bile salts, or vitamin D analogs tested. However, a planar ligand previously reported to activate AHRs, 6-formylindolo-[3,2-b]carbazole [72] robustly activated the *Ciona *VDR/PXR. Weaker activation was also achieved with two other planar ligands, carbamazepine (an anti-epilepsy medication) and *n*-butyl *p*-aminobenzoate (a compound that also activates African clawed frog PXRs [7,12,49], Western clawed frog PXR (this report), as well as several other PXRs [12]). A preliminary three-point pharmacophore indicates a relatively planar pharmacophore for *Ciona *VDR/PXR consisting of an off-center hydrogen bond acceptor flanked by two hydrophobic regions. This pharmacophore is different compared with those from the other six species described herein in that it is more restrictive. Intrinsic disorder analysis also suggests that *Ciona *VDR/PXR is more similar to PXR in the LBD than to VDR. The added disorder in the LBD (relative to VDR) may make it able to adapt to different ligands.

Our phylogenetic analysis, including reconstruction of ancestral sequences by maximum likelihood, is consistent with (although certainly does not prove) the hypothesis that VDR represents the ancestral NR1I gene [51,73]. Comparison of ligand-binding residue positions between extant and reconstructed ancestral sequences also suggests that PXR may represent the gene ancestral to extant mammalian CARs and PXRs. Identification of additional NR1I receptors in basal vertebrates, chordate invertebrates other than *Ciona*, reptiles, and basal mammals will be valuable in developing a more complete evolutionary history in future studies.

These results are consistent with the natural ligands of *Ciona *VDR/PXR being markedly different than those of vertebrate VDRs or PXRs. It is perhaps noteworthy that the most potent and efficacious activator of *Ciona *VDR/PXR discovered in this study (6-formylindolo-[3,2-b]carbazole) is also a potent activator of vertebrate AHRs [72,74]. Studies of invertebrate AHRs reveal markedly different ligand selectivity compared to vertebrate AHRs [75] and also roles of the AHR system in invertebrate development [76,77]. Future studies will be aimed at identifying possible endogenous ligands of the *Ciona *VDR/PXR and other *Ciona *NRs; however, if the ligands for the *Ciona *receptor are exogenous, they may ultimately be difficult to uncover.

## Conclusion

In contrast to other nuclear hormone receptors, we have demonstrated *in vitro *that PXRs show significant differences in ligand specificity across species. Further, by pharmacophore analysis, certain PXRs share similar molecular requirements, suggestive of functional overlap. The PXR of the Western clawed frog has diverged considerably in ligand selectivity from fish, bird, and mammalian PXRs. The LBD of zebrafish PXR is smaller than those of the mammals and is activated by a more limited range of compounds. Even more restricted is the small set of ligands found to activate *Ciona *VDR/PXR. Taken in sum, the ligand selectivity of PXR is surprisingly species dependent, and has undergone an ever expanding role in the progression of evolution from pre-chordates to fish to mammals and birds. The combined results suggest that using a combination of *in vitro *and computational methods we can qualitatively explain the unusual evolutionary history shaping the ligand selectivity of PXRs and this may be applicable to other proteins.

## Methods

### Chemicals

The sources of the chemicals were as follows: *n*-butyl-*p*-aminobenzoate, *n*-propyl-*p*-hydroxybenzoate, nifedipine, rifampicin (Sigma-Aldrich, St. Louis, MO, USA); 5α-cholanic acid-3α,7α,12α-triol (allocholic acid; Toronto Research Chemical, Inc., North York, ON, Canada); Nuclear Receptor Ligand Library (76 compounds known as ligands of various nuclear hormone receptors; BIOMOL). 5α-cyprinol sulfate (5α-cholestan-3α,7α,12α,26-tetrol-27-sulfate) was isolated from Asiatic carp (*Cyprinus carpio*) bile [78], 5β-scymnol sulfate (5β-cholestan-3α,7α,12α,24,26-pentol-27-sulfate) was isolated from the bile of Spotted eagle ray (*Aetobatus narinari*) bile, and 5β-cholestan-3α,7α,12α-triol-27-oic acid, taurine conjugated was isolated from the bile of the American alligator (*Alligator mississippiensis*). Bile salts were purified by extraction and Flash column chromatography. Bile alcohol sulfates were chemically deconjugated using a solution of 2,2-dimethoxypropane:1.0 N HCl, 7:1 v/v, and incubating 2 hours at 37°C, followed by the addition of water and extraction into ether. Completeness of deconjugation and assessment of purity was performed by thin-layer chromatography using known standards. Other than those described above, steroids and bile salts were obtained from Steraloids (Newport, RI, USA).

### Animals

*Xenopus tropicalis *frogs were obtained from NASCO (Fort Atkinson, WI, USA). All animal studies were performed in conformity with the Public Health Service Policy on Humane Care and Use of Laboratory Animals, incorporated in the Institute for Laboratory Animal Research Guide for Care and use of Laboratory Animals. All vertebrate animal studies were approved by the University of Pittsburgh Institutional Animal Care and Use Committees (approval number 0601348) or Committee on Animal Studies of the University of California, San Diego.

### Cloning and molecular biology

The LBD of *Xenopus tropicalis *(Western clawed frog) PXR (xtPXR) was cloned by PCR from RNA extracted from ovary of an adult female frog. *Ciona *VDR/PXR was cloned from cDNA fragments ciem829d05 and cilv048e18 provided by Professor Yuji Kohara (Center for Genetic Resource Information, National Institute of Genetics, Research Organization of Information and Systems, Mishima, Japan) and Professor Noriyuki Satoh (Kyoto University, Kyoto, Japan), with analysis of the cDNA clones supported by Grant-in-aid for Scientific Research on Priority Area "Genome" of Ministry of Education, Culture, Sports, Science and Technology, Japan. The LBD of xtPXR (residues 103–390) and *Ciona *VDR/PXR (residues 57–391) were inserted into the pM2-GAL4 vector to create a GAL4/LBD chimera suitable for study of ligand activation [17].

### Cell culture and functional assays

The creation of a HepG2 (human liver) cell line stably expressing the human Na^+^-taurocholate cotransporter (NTCP) has been previously reported [17]. HepG2-NTCP cells were grown in modified Eagle's medium-α containing 10% fetal bovine serum and 1% penicillin/streptomycin (Invitrogen, Carlsbad, CA, USA). Plasmids containing cDNAs for 8 PXRs from 7 species (human, mouse, rat, rabbit, chicken [also called chicken X receptor, CXR], African clawed frog [also termed *Xenopus laevis *benzoate X receptors α and β, BXRα and BXRβ ], zebrafish), as well as the reporter constructs tk-UAS-Luc and CYP3A4-PXRE-Luc, and 'empty' vectors pCDNA, PsG5, and PM2 were generously provided by SA Kliewer, JT Moore, and LB Moore (GlaxoSmithKline, Research Triangle Park, NC, USA). The expression vectors were either full-length receptors (i.e., containing both a DBD and LBD; human, mouse, rat, rabbit, and chicken PXRs) or GAL4/PXR chimeras that contain only the LBD of the PXR receptor (BXRα, BXRβ, *Xenopus tropicalis *PXR, and zebrafish PXR). For the full-length expression vectors, the reporter plasmid was CYP3A4-PXRE-Luc, a construct that contains a promoter element from CYP3A4 (recognized by PXR DBDs) driving luciferase expression. For the GAL4/LBD expression constructs, the reporter plasmid was tk-UAS-Luc, which contains GAL4 DNA binding elements driving luciferase expression. The following transfection ratios of reporter, receptor, and β-galactosidase plasmids were used (ng/well): human, mouse, rabbit, and rat PXRs – 25/2.7/20; chicken PXR – 10/1/20; *Xenopus tropicalis *PXR, zebrafish PXR, and *Ciona *VDR/PXR – 75/50/20.

The PXR activation assay was a luciferase-based reporter assay [17,45]. On day 1, 30,000 cells/well were seeded onto 96-well white opaque plates (Corning-Costar, Corning, NY, USA). On day 2, cells were transfected using the calcium phosphate precipitation method with expression vector or 'empty' control vector and luciferase reporter plasmid. On day 3, the cells were washed and then incubated with medium containing charcoal-dextran treated fetal bovine serum (Hyclone, Logan, UT, USA) and drugs or vehicle. On day 4, the cells were washed and the medium replaced with serum-free medium. Cells were washed with Hanks' buffered salt solution and then exposed to 150 μL lysis buffer (Reporter Lysis Buffer, Promega). Separate aliquots were taken for measurement of β-galactosidase activity (Promega) and luciferase activity (Promega Steady-Glo luciferase assay).

Activation of receptor by ligand was compared to receptor exposed to identical conditions without ligand ('vehicle control'). In general, dimethyl sulfoxide (Sigma) was used as vehicle and was adjusted to be 0.5% (v/v) in all wells. A control was also run with transfection of 'empty' vector (i.e., lacking the receptor cDNA) and reporter vector to control for activation of reporter vector by endogenous receptor(s). In experiments with a variety of activators, activation by endogenous receptors was not seen.

To facilitate more reliable cross-species comparisons, complete concentration-response curves for ligands were determined in the same microplate as determination of response to a maximal activator. This allows for determination of relative efficacy, ε defined as the maximal response to test ligand divided by maximal response to a reference maximal activator (note than ε can exceed 1). The following maximal activators and their concentrations were as follows: human PXR – 10 μM rifampicin; mouse and rat PXRs – 10 μM pregnenolone 16α-carbonitrile; rabbit PXR – 50 μM 5β-pregnane-3,20-dione; chicken PXR – 20 μM nifedipine; *Xenopus tropicalis *PXR – *n*-propyl-*p*-hydroxybenzoate 50 μM; zebrafish PXR – 20 μM 5α-androstan-3α-ol; and *Ciona *VDR/PXR – 20 μM 6-formylindolo-[3,2-*b*]carbazole. All comparisons to maximal activators were done within the same microplate. Luciferase data were normalized to the internal β-galactosidase control and represent means ± SD of the assays. Concentration-response curves were fitted using Kaleidagraph software (Synergy Software, Reading, PA, USA). In combining data from multiple experiments, the pooled variance was calculated by the formula s_pooled _= {[(n_1_-1)s_1_^2 ^+ (n_2_-1)s_2_^2 ^+ ... + (n_k_-1)s_k_^2^]/[N-k]}^-1/2^, where there are N total data points among k groups, with n replicates in the i^th ^group.

### Toxicity assays in HepG2 cells

To test for cytotoxicity, two assays that have been well-validated in HepG2 cells were used: 3-(4,5-dimethylthiazol-2-yl)-2,5-diphenyltetrazolium bromide (MTT) reduction and alamar blue reduction. Both assays sensitively measure the ability of viable cells to metabolize the parent compound to a metabolite that can be detected by spectrophotometry or fluorometry [79]. HepG2 cells were seeded at a density of 20,000 cells/well (100 μL per well) into clear 96-well microplates (for the MTT assay) or black, opaque 96-well plates (for the alamar blue assay) and grown for 24 hours. The next day, 100 μL solutions of drug concentrations or vehicle controls in cell growth medium at twice the intended final concentration were added to the cells (final volume 200 μL). The cells were again incubated for 24 hr. For the MTT assays, MTT (In vitro toxicology assay kit, MTT-based; Sigma, St. Louis, MO, USA) was dissolved at 5 mg/mL in warm cell growth medium. 20 μL of this solution was added to the cells (total volume 220 μL), and the plates incubated for another 4 hrs. After incubation, the supernatant was removed and 50 μL of solubilization buffer provided in the Sigma kit with 0.5% DMSO was added. DMSO was added to ensure total solubility of the formazan crystals. Plates were shaken for 2 min, and the absorbance recorded at 590 nm. The percent viability was expressed as absorbance in the presence of test compound as a percentage of that in the vehicle control (with subtraction of background absorbance).

For the alamar blue assays, alamar blue stock solution (Biosource International; Camarillo, CA, USA) was diluted 1:1 with cell growth medium and 50 μL of this was added to each well, yielding a final concentration of 10% alamar blue (total volume 250 μL). The plates were exposed to an excitation wavelength of 530 nm, and the emission at 590 nm was recorded to determine whether any of the test drug concentrations fluoresce at the emission wavelength. Plates were returned to the incubator for 5 hr and the fluorescence was measured again. The percent viability was expressed as fluorescence counts in the presence of test compound as a percentage of that in the vehicle control (with subtraction of background fluorescence). Drug concentrations that cause > 30% loss of cell viability in the MTT assay or > 15% loss of cell viability in the alamar blue assay were not used in the determination of concentration-response curves for activation of PXRs.

### *In silico *modelling – Catalyst™

Pharmacophore modelling was performed as described previously [45,54]. Briefly, computational molecular modeling studies were carried out using Discovery Studio 1.7 Catalyst™ (Accelrys, San Diego, CA) running on a Sony Vaio with Intel Centrino processor. Pharmacophore models attempt to describe the arrangement of key features that are important for biological activity. Briefly, the Catalyst™ models were employed to generate hypotheses. Molecules were imported from sdf files, the 3-D molecular structures were produced using up to 255 conformers with the Best conformer generation method, allowing a maximum energy difference of 20 kcal/mol. Hypogen PXR pharmacophores for each species were generated with Catalyst™ using the 16 molecules in Table 4. Molecules highlighted in **bold type **were used for training as they are common to all species – molecules with no effect were given the arbitrary EC_50 _value of 10,000 μM (10 mM).

Ten hypotheses were generated using these conformers for each of the molecules and the EC_50 _values, after selection of the following features: hydrophobic, hydrogen bond acceptor, and hydrogen bond donor with up to 4 excluded volumes. After assessing all ten generated hypotheses, the hypothesis with the lowest energy cost was selected for further analysis as this possessed features representative of all the hypotheses and had the lowest total cost. The quality of the structure activity correlation between the estimated and observed activity values was estimated by means of an *r *value. Additionally 6-formylindolo-[3,2-*b*]carbazole was aligned with carbamazepine and *n*-butyl-*p*-aminobenzoate with the HIPHOP alignment to ascertain the pharmacophore for *Ciona *VDR/PXR.

### Phylogenetic analysis and ancestral sequence reconstruction

The following sequences were used for phylogenetic analysis and ancestral sequence reconstruction (some links are from the Ensembl database [80]): human VDR [GenBank: NM_00376], chimpanzee VDR [Ensembl:ENSPTRT00000009010], rhesus monkey VDR [Ensembl:ENSMMUT00000009414], cow VDR [Ensembl:ENSBTAT00000021832], dog VDR [Ensembl:ENCAFT00000014497], mouse VDR [GenBank: NM_008504], rat VDR [GenBank: NM_009504], chicken VDR [GenBank: AF011356], Japanese quail VDR [GenBank: U12641], *Xenopus laevis *VDR [GenBank: U91849], Atlantic salmon (*Salmo salar*) VDR [GenBank: AJ780914], fugu VDR [Ensembl:NEWSINFRUT00000138841], bastard halibut (*Paralichthys olivaceus*) VDR [GenBank: AB037674], zebrafish VDR [GenBank: AF164512], medaka VDR [Ensembl:ENSORLT00000001311], stickleback fish (*Gastrosteus aculeatus*) VDR [Ensembl:ENSGACT00000006308], sea lamprey VDR [GenBank: AY249863], *Ciona intestinalis *VDR/PXR [GenBank: BR000137], human CAR [GenBank: NM_005122], chimpanzee CAR [ENSPTRT00000002884], rhesus CAR [GenBank: AY116212], cow CAR [Ensembl:ENSBTAT00000012145], dog CAR [Ensembl:ENSCAFT00000020528], pig CAR [GenBank: AB214979], Baikal seal (*Phoca sibirica*) CAR [GenBank: AB109553], Northern fur seal (*Callorhinus ursinus*) CAR [GenBank: AB109554], mouse CAR [GenBank: NM_009803], rat CAR [GenBank: NM_022941], pig CAR [GenBank: AB214979], opossum CAR [Ensembl:ENSMODT00000006393], human PXR [GenBank: AF061056], chimpanzee PXR [ENSPTRT00000028510], rhesus monkey PXR [GenBank: AF454671], cow PXR [Ensembl:ENSBTAT00000026059], mouse PXR [AF031814], rat PXR [GenBank: NM_052980], rabbit PXR [GenBank: AF188476], opossum PXR [Ensembl:ENSMODT00000023109], chicken PXR [GenBank: AF276753], *Xenopus laevis *PXRα [GenBank: BC041187], *Xenopus laevis *PXRβ [GenBank: AF305201], *Xenopus tropicalis *PXR [Ensembl:ENSXETT00000039109], fugu PXR [Ensembl:NEWSINFRUT00000171584], medaka PXR [Ensembl:ENSORLT00000022473], *Tetraodon nigriviridis *PXR [Ensembl:GSTENT00026021001], zebrafish PXR [GenBank: AF454674, GenBank: AF502918], domestic silkworm (*Bombyx mori*) ecdysone receptor [GenBank: L35266], ixotid tick (*Amblyomma americanum*) ecdysone receptor [GenBank: AF020187], and purple sea urchin (*Strongylocentrotus purpuratus*) liver X receptor [GenBank: XM_774904]. Sequences were aligned using ClustalW [81] and Tcoffee software [82] and manually adjusted as needed.

Phylogeny was inferred by maximum likelihood using PHYML software [83,84], assuming a WAG protein model and a 4-category discrete gamma distribution of among-site rate variation. To estimate support, 100 bootstrap replicates were analyzed. Ancestral protein sequences of AncR1, AncR2, and AncR3 (see nodes in Figure 4) were inferred by maximum likelihood using PAML 3.15 software [85,86] on the maximum likelihood phylogeny of 49 amino acid sequences of extant VDRs, PXRs, and CARs (see Figure 4). For ancestral reconstruction, the JTT+G model (supported with 100% posterior probability in the Bayesian analysis) was assumed. Residues that interacted closely with ligands in published X-ray crystallographic structures of human VDR [58-60], rat VDR [61,62], zebrafish VDR [63], human PXR [29-34], human CAR [64,65], and mouse CAR [66] were identified and designated in Figure 5 and Additional files 7 and 8 as 'ligand-binding residues'.

### Calculation of protein structural disorder

Intrinsic disorder prediction of protein sequences were performed using the PONDR VL3H algorithm [68,87]. The disorder calculations for each amino acid residue are available as Additional file 10.

## Abbreviations

Benzoate X receptor, BXR; constitutive androstane receptor, CAR; chicken X receptor, CXR; DNA-binding domain, DBD; ligand-binding domain, LBD; nuclear hormone receptor, NR; 3-(4,5-dimethylthiazol-2-yl)-2,5-diphenyltetrazolium bromide, MTT; pregnane X receptor, PXR; 2,3,7,8-tetrachlorodibenzo-p-dioxin, TCDD; vitamin D receptor, VDR.

## Authors' contributions

SE performed the molecular modeling and protein disorder studies and helped draft the manuscript. EJR performed the molecular biology and assisted with the functional assays. LRH purified bile salts from animal bile to use as PXR ligands. MDK conceived of the study, performed most of the functional assays, and drafted the manuscript. All authors contributed to, read, and approved the final manuscript.

## Supplementary Material

Additional file 1 Sequence alignment of nine PXRs and the *Ciona *VDR/PXR. Sequence alignment of the DNA-binding and ligand-binding domains of PXRs and the *Ciona intestinalis *VDR/PXR Click here for file

Additional file 2 Table of activation data for human and zebrafish PXRs by bile salts. Summary of concentration-response data for activation of human and zebrafish PXRs by bile salts Click here for file

Additional file 3 Comparison of bile salt activation of human and zebrafish PXRs. Comparison of bile salt synthetic pathways for humans and zebrafish, indicating which bile salts and intermediates activate human and zebrafish PXRs. Click here for file

Additional file 4 Table summary of statistics of the pharmacophore models. Summary of the statistics for the pharmacophore models of activation of PXRs. Click here for file

Additional file 5 Additional data for zebrafish PXR, *Xenopus tropicalis *PXR, and *Ciona intestinalis *VDR/PXR. Summary of data for screening of compounds as possible activators for zebrafish PXR, *Xenopus tropicalis *PXR, and the *Ciona *VDR/PXR. Click here for file

Additional file 6 HIPHOP model for *Ciona *VDR/PXR. HIPHOP alignment of carbamazepine, 6-formylindolo-[3,2-*b*]carbazole, and *n*-butyl-*p*-aminobenzoate as activators of *Ciona *VDR/PXR. Click here for file

Additional file 7 Reconstructed ancestral sequences. Detailed data for the reconstruction of ancestral sequences. Click here for file

Additional file 8 Comparison of ligand-binding residues between extant and reconstructed sequences. Conservation of ligand-binding residues in extant and reconstructed PXR sequences. Click here for file

Additional file 9 Intrinsic disorder summary for PXRs, VDRs, and CARs. Summary data for intrinsic disorder predictions for PXR, VDR, and CAR sequences. Click here for file

Additional file 10 Intrinsic disorder data for PXRs, VDRs, and CARs. Intrinsic disorder data for each amino acid residue of PXR, VDR, and CAR sequences analyzed. Click here for file
