# Supplementary material for: Evolution of pharmacologic specificity in the pregnane X receptor
Source: BMC Evol Biol. 2008 Apr 2;8:103. doi: 10.1186/1471-2148-8-103 (PMC2358886; doi:10.1186/1471-2148-8-103)
Supplement: Additional file 1 — Sequence alignment of nine PXRs and the Ciona VDR/PXR. Sequence alignment of the DNA-binding and ligand-binding domains of PXRs and the Ciona intestinalis VDR/PXR [file 1471-2148-8-103-S1.pdf]

A. DNA-binding domain

```
PXR_human          QICRVCGDKATGYHFNVMTCEGCKGFFRRAMKRNARLRCPFRKGACEITRKTRRQCQACR
PXR_mouse          QICRVCGDKANGYHFNVMTCEGCKGFFRRAMKRNVRLRCPFRKGTCEITRKTRRQCQACR
PXR_rat            QICRVCGDKANGYHFNVMTCEGCKGFFRRAMKRNVRLRCPFRKGTCEITRKTRRQCQACR
PXR_rabbit         QTCRVCGDKANGYHFNVLTCEGCKGFFRRTVKRNARLRCPFRKGACEITRKTRRQCQACR
PXR_chicken        KVCAVCGDRATGYHFHVMSCEGCKGFFRRSILKGVHFTCPF-TRSCPITKAKRRQCQACR
BXR-alpha          KICRACGDRATGYHFNAMTCEGCKGFFRRAMKRNLRLSCPF-QNSCVINKSNRRHCQACR
BXR-beta           KICRACGDRATGYHFNAMTCEGCKGFFRRAMKRNLRLSCPF-QNSCVINKNNRRHCQACR
PXR_xentrop        KICRACGDRATGYHFNAMTCEGCKGFFRRAMKRKLQLSCPF-QNSCVINKSNRRHCQACR
PXR_zebrafish      KICQVCGDKSTGYHFNAMTCEGCKGFCRRAMKRPAQLCCPF-QSACVITKSNRRQCQSCR
VDR_PXR_Ciona      ------------MHFGAITCEGCKGFFRRSVKKNASFSCAF-EKKCEINKNNRKHCQACR

PXR_human          LRKCLESGM
PXR_mouse          LRKCLESGM
PXR_rat            LRKCLESGM
PXR_rabbit         LRKCLESGM
PXR_chicken        LQKCLDVGM
BXR-alpha          LKKCLDIGM
BXR-beta           LKKCLDIGM
PXR_xentrop        LKKCLDIGM
PXR_zebrafish      LQKCLSIGM
VDR_PXR_Ciona      FNACLAAGM
```

B. Ligand-binding domain

```
                         Helix-1_________
PXR_human      141 QGLTEEQRMMIRELMDAQMKTFDTTFSHFKNFRLPGVLSSGCELPESLQAP-SREEA-AK
PXR_mouse          QGLTEEQQALIQELMDAQMQTFDTTFSHFKDFRLPAVFHSGCELPEFLQAS-LLEDP-AT
PXR_rat            QGLTEEQQALIQELMDAQMQTFDTTFSHFKDFRLPAVFHSDCELPEVLQAS-LLEDP-AT
PXR_rabbit         QGLTGEQRMIIEELMDAQMKTFDTTFSHFKNFRLPEVLGSGCEIPESLQAL-TEEEA-GR
PXR_chicken        GGLTAEQQELISILIAAHKRTFDSSFSQFQHYQPAVRLC----IPGPCSQS-PPGPG-VP
BXR-alpha          ASLTPEQQHFLTQLVGAHTKTFDFNFTFSKNFRPIRRSSDPTQEPQAT------------
BXR-beta           ASLTPEQQHFITELVEAHTKTFDFNFTFFKNFRPIRRSPDPTQDPQAT------------
PXR_xentrop        TSLTPEQQHFVTELAGAHTKTFDFNFSSFKNFRPIKRSSDPTQDPQAT------------*
PXR_zebrafish      VTLTPQQEAVIQELLNAHKKTFDMTCAHFSQFRPLDRDQKSVSESSPLTNG-SWIDH-RP
VDR_PXR_Ciona      TRMTMDEKLLVKTLLKGHRDSYDFAYVEYDTFRGREDGQQEIGNNTENPNG---LDA-AT

                                                                  Helix-3_____
PXR_human      199 WSQVRKDLCSLKVSLQL-RGEDGSVWNYKPP-----ADSGGKEIFSLLPHMADMSTYMFK
PXR_mouse          WSQIMKDRVPMKISLQL-RGEDGSIWNYQPP-----SKSDGKEIIPLLPHLADVSTYMFK
PXR_rat            WSQIMKDSVPMKISVQL-RGEDGSIWNYQPP-----SKSDGKEIIPLLPHLADVSTYMFK
PXR_rabbit         WRQIQEELGTMKLSLQL-RGEDGSVWNYTPP-----ADRSGKKLFSLLPHLADMSTYMFK
PXR_chicken        SASLSPQLDCLDEDVL------------------------PDVFSILPHFADLSTFMIQ
BXR-alpha          ---------------------------------------SSEAFLMLPHISDLVTYMIK
BXR-beta           ---------------------------------------SSEAFLMLPHISDLFTYMLK
PXR_xentrop        ---------------------------------------SSGAFLMLPHISDLITYMIK
PXR_zebrafish      IAEDPMQWVFNPTSLSS-SSSSYQSLDNKEK-----KHFKSGN-FSSLPHFTDLTTYMIK
VDR_PXR_Ciona      AVEAQSTTEDSGKQLHL-MLLFQHFLPIYP------FSFDPKA-KQLFQHFCDIMTWGIR

                   Helix-3          Helix-4     Helix-5
PXR_human      253 GIISFAKVISYFRDLPIEDQISLLKGAAFELCQLRFNTVFNAET-GTWECGR--LSY---C
PXR_mouse          GVINFAKVISYFRDLPIEDQISLLKGATFEMCILRFNTMFDTET-GTWECGR--LAY---C
PXR_rat            GVINFAKVISHFRELPIEDQISLLKGATFEMCILRFNTMFDTET-GTWECGR--LAY---C
```

```
PXR_rabbit         GIINFAKVISYFRDLPIEDQISLLKGATLELCLLRFNTVFNAET-GTWECGR--LSY---C
PXR_chicken        QVIKFAKEIPAFRGLPIDDQISLLKGATLGICQIQFNTVFNEET-NAWECGQ--HCF---T
BXR-alpha          GIISFAKMLPYFKSLDIEDQIALLKGSVAEVSVIRFNTVFNPDT-NTWECGP--FTY---D
BXR-beta           GVISFAKMLPYFRSLAIEDQIALLKGSVLEVCVIRFNRMFNPKT-NTWECGA--FTY---N
PXR_xentrop        GVISFAKMLPYFKSLNIEDQIALLKGSVVEVCVIRFNTMFVPET-NSWECGP—-ITY---N
PXR_zebrafish      NVINFGKTLTMFRALVMEDQISLLKGATFEIILIHFNMFFNEVT-GIWECGP--LQY---C
VDR_PXR_Ciona      KVIDYCKGIPQFVQLSIVDQIVLLRGGCLEMLVLRSYFAFSCNE-NKYMSDK--FQY---K

                                 Helix-7             Helix-8           Helix-9
PXR_human      308 LEDT-AGGF----QQLLLEPMLKFHYMLKKLQLHEEEYVLMQAISLFSPDRPGVLQHRVV
PXR_mouse          FEDP-NGGF----QKLLLDPLMKFHCMLKKLQLHKEEYVLMQAISLFSPDRPGVVQRSVV
PXR_rat            FEDP-NGGF----QKLLLDPLMKFHCMLKKLQLREEEYVLMQAISLFSPDRPGVVQRSVV
PXR_rabbit         VEDP-EGGF----QQLLVDPLLKFHYMLKKLQLHKEEYVLMQAISLFSPDRPGVVQREVV
PXR_chicken        IKDGALAGF----QQIYLEPLLKFHISLKKLRLHEAEYVLLVAMLLFSPDHASVTQRDFI
BXR-alpha          TEDMFLAGF----RQLFLEPLVRIHRMMRKLNLQSEEYAMMAALSIFASDRPGVCDWEKI
BXR-beta           ADDMTMAGF----SQQFLEPLLRIHCMMTKLNLESEAYALMATMALFSSDRPGVSDCEKI
PXR_xentrop        TEDMTMAGF----RQLFLEPLLRMHRMMRKLNLHNEEYALMAAMALFASDRPGVQDCKKI
PXR_zebrafish      MDDAFRAGF----QHHLLDPMMNFHYTLRKLRLHEEEYVLMQALSLFSPDRPGVTDHKVI
VDR_PXR_Ciona      PSDFLQAGG----NKEFVEKYNSLHIRMRKMKLQVEEICLLLALVLFSPDRPGLEDQAKV

                      Helix-9                   Helix-10________________
PXR_human      363 DQLQEQFAITLKSYIECNR-PQPAHRFLFLKIMAMLTELRSINAQHTQRLLRIQDIHPFA
PXR_mouse          DQLQERFALTLKAYIECSR-PYPAHRFLFLKIMAVLTELRSINAQQTQQLLRIQDSHPFA
PXR_rat            DQLQERFALTLKAYIECSR-PYPAHRFLFLKIMAVLTELRSINAQQTQQLLRIQDTHPFA
PXR_rabbit         DQLQERFAITLKAYIECSR-PQPTHRFLFLKIMAVLTELRTINAQHTQRLLRIQDTHPFA
PXR_chicken        DQLQEKVALTLKSYIDHRH-PMPEGRFLYAKLLLLLTELQTLKMENTRQILHIQDLSSM-
BXR-alpha          QKLQEHIALTLKDFIDSQRPPSLQNRLLYPKIMECLTELRTVNDIHSKQLLEIWDIQPDA
BXR-beta           QNLQEHIALMLKAFIESHRPPSPQNRLLYPKIMECLTELRTINDIHSKQLMEIWDIQPDV
PXR_xentrop        QNLQEHIALMLKRYIECQRPLSPQNRLLYPKIMECLTELRTVNDIHSKQLLEIWDIQPDA
PXR_zebrafish      DRNQETLALTLKTYIEAKR-NGPEKHLLFPKIMGCLTEMRSMNEEYTKQVLKIQDMQPEV
VDR_PXR_Ciona      EQMQDCVANTLQAYEYTHK-PPNESSFLQARTMYCLPILRTINMLFAQNIMSLQTNEKDM

PXR_human      422 T-PLMQELFGITGS
PXR_mouse          T-PLMQELFSSTDG
PXR_rat            T-PLMQELFSSTDG
PXR_rabbit         T-PLMRELFSTTDD
PXR_chicken        T-PLLSEIIS
BXR-alpha          T-PLMREVFGSPE
BXR-beta           T-PLMREVFGSLNE
PXR_xentrop        T-PLLREVFGSHND
PXR_zebrafish      S-PLWLEIISKDT
VDR_PXR_Ciona      N-PLILEVNNSADDED
```

Additional file 1: Sequence alignment of (A) DNA-binding domain and (B) ligand-binding domain of PXRs from diverse vertebrate species and chordate invertebrate *Ciona intestinalis*. The ligand-binding domain is annotated with the α-helices [1]. Residues highlighted in **bold** are residues shown to contact ligand in crystal structures of human PXR bound to SR121813 [2, 3], hyperforin [4], rifampicin [5], T-0901317 [6], and 17β-estradiol [7]. Accession numbers are: human PXR [Genbank:AF061056], mouse PXR [Genbank:AF031814], rat PXR [Genbank:AF151377], rabbit PXR [Genbank:AF188476], chicken PXR [Genbank:AF276753], *Xenopus laevis* BXRα [Genbank:BC041187], *Xenopus laevis* BXRβ [Genbank:AF305201], *Xenopus tropicalis* PXR [Ensembl, http://www.ensembl.com:ENSXETT00000039109], zebrafish PXR [Genbank:AF454673,Genbank:AF502918], and *Ciona intestinalis* VDR/PXR [Genbank:BR000137].

References

1. Moore LB, Maglich JM, McKee DD, Wisely B, Willson TM, Kliewer SA, Lambert MH, Moore JT: **Pregnane X receptor (PXR), constitutive androstane receptor (CAR), and benzoate X receptor (BXR) define three pharmacologically distinct classes of nuclear receptors**. *Mol Endocrinol*, 2002, **16(5):**977-986.
2. Watkins RE, Davis-Searles PR, Lambert MH, Redinbo MR: **Coactivator binding promotes the specific interaction between ligand and the pregnane X receptor**. *J Mol Biol*, 2003, **331(4):**815-828.
3. Watkins RE, Wisely GB, Moore LB, Collins JL, Lambert MH, Williams SP, Willson TM, Kliewer SA, Redinbo MR: **The human nuclear xenobiotic receptor PXR: structural determinants of directed promiscuity**. *Science*, 2001, **292(5525):**2329-2333.
4. Watkins RE, Maglich JM, Moore LB, Wisely GB, Noble SM, Davis-Searles PR, Lambert MH, Kliewer SA, Redinbo MR: **2.1 Å crystal structure of human PXR in complex with the St. John's wort compound hyperforin**. *Biochemistry*, 2003, **42(6):**1430-1438.
5. Chrencik JE, Orans J, Moore LB, Xue Y, Peng L, Collins JL, Wisely GB, Lambert MH, Kliewer SA, Redinbo MR: **Structural disorder in the complex of human PXR and the macrolide antibiotic rifampicin**. *Mol Endocrinol*, 2005, **19(5):**1125-1134.
6. Xue Y, Chao E, Zuercher WJ, Willson TM, Collins JL, Redinbo MR: **Crystal structure of PXR-T1317 complex provides a scaffold to examine the potential for receptor antagonism**. *Bioorg Med Chem*, 2007, **15(5):**2156-2166.
7. Xue Y, Moore LB, Orans J, Peng L, Bencharit S, Kliewer SA, Redinbo MR: **Crystal structure of PXR-estradiol complex provides insights into endobiotic recognition**. *Mol Endocrinol*, 2007, **21(5):**1028-1038.
