# Supplementary material for: Evolution of pharmacologic specificity in the pregnane X receptor
Source: BMC Evol Biol. 2008 Apr 2;8:103. doi: 10.1186/1471-2148-8-103 (PMC2358886; doi:10.1186/1471-2148-8-103)
Supplement: Additional file 2 — Table of activation data for human and zebrafish PXRs by bile salts. Summary of concentration-response data for activation of human and zebrafish PXRs by bile salts [file 1471-2148-8-103-S2.pdf]

Additional file 2: Activation of human and zebrafish PXRs by bile salts

Cmp. #	Compound	hPXR Activity	hPXR Efficacy	zfPXR Activity	zfPXR Efficacy	Toxicity
BI1	5β-Cholan-3α,7α,12α,24-tetrol (5β-petromyzonol)	None		None		None
BI2	5β-Cholanic acid-3α,6α-diol (hyodeoxycholic acid)	4.42	0.17	None		None
BI3	5β-Cholanic acid-3α,6α-diol *N*-(2-sulfoethyl)-amide (taurohyodeoxycholic acid)	4.72	0.39	None		200
BI4	5β-Cholanic acid-3α,6β-diol (murideoxycholic acid)	None		None		None
BI5	5β-Cholanic acid-3α,7α-diol (chenodeoxycholic acid)	None		None		200
BI6	5β-Cholanic acid-3α,7α-diol *N*-(carboxymethyl)-amide (glycochenodeoxycholic acid)	None		None		None
BI7	5β-Cholanic acid-3α,7α-diol *N*-(2-sulfoethyl)-amide (taurochenodeoxycholic acid)	3.98	0.5	None		None
BI8	5β-Cholanic acid-3α,12α-diol (deoxycholic acid)	4.30	0.19	None		200
BI9	5β-Cholanic acid-3α,12α-diol *N*-(carboxymethyl)-amide (glycodeoxycholic acid)	None		None		None
BI10	5β-Cholanic acid-3α,12α-diol *N*-(2-sulfoethyl)-amide (taurodeoxycholic acid)	None		None		None
BI11	5β-Cholanic acid-3α-ol (lithocholic acid)	5.00	0.15	None		200
BI12	5β-Cholanic acid-3α-ol *N*-(carboxymethyl)-amide (glycolithocholic acid)	4.79	0.49	None		200
BI13	5β-Cholanic acid-3α-ol *N*-(2-sulfoethyl)-amide (taurolithocholic acid)	4.70	0.15	None		200
BI14	5β-Cholanic acid-3α-ol-7,12-dione (7,12-diketolithocholic acid)	4.45	0.31	None		None
BI15	5β-Cholanic acid-3α-ol-7-one (7-ketolithocholic acid)	4.67	0.58	None		None
BI16	5β-Cholanic acid-3α-ol-12-one (12-ketolithocholic acid)	4.50	0.86	None		200
BI17	5β-Cholanic acid-3α,6α,7β-triol (ω-muricholic acid)	None		None		None
BI18	5β-Cholanic acid-3α,6β,7α-triol (α-muricholic acid)	4.25	0.79	None		None
BI19	5β-Cholanic acid-3α,6β,7β-triol (β-muricholic acid)	None		None		None
BI20	5β-Cholanic acid-3α,7α,12α-triol (cholic acid)	4.94	0.56	None		None
BI21	5β-Cholanic acid-3α,7α,12α-triol *N*-(carboxymethyl)-amide (glycocholic acid)	None		None		None
BI22	5β-Cholanic acid-3α,7α,12α-triol *N*-(2-sulfoethyl)-amide (taurocholic acid)	None		None		200
BI23	5β-cholestan-3α,7α,12α-triol	None		None		20
BI24	20α-hydroxycholesterol	None		None		None
BI25	Cholesterol	None		None		None

BI26	23-Nordeoxycholic acid	4.79	0.5	None		None
BI27	23-Norcholic acid	4.02	0.49	None		None
BI28	5α-Cholan-7α,12α,-24-triol-3-one	None		None		None
BI29	5α-Cholan-3α,7α,12α,-24-tetrol (5α-petromyzonol)	None		None		50
BI30	5α-Cholan-3α,7α,12α-triol-24-sulfate (5α-petromyzonol sulfate)	4.55	1.02	None		None
BI31	5α-Cholanic acid-3α,7α,12α-triol (allocholic acid)	None		None		200
BI32	5α-Cholan-7α,12α-diol-3-one-24-sulfate	None		None		None
BI33	5α-Cholanic acid-7α,12α-diol-3-one	4.92	0.26	None		None
BI34	5β-Cholestan-3α,7α,12α,24,26-pentol-27-sulfate (5β-scymnol sulfate)	4.31	0.39	4.35	0.13	200
BI35	5β-Cholestan-3α,7α,12α,24,26-hexol (5β-scymnol)	None		None		100
BI36	5β-Cholestan-3α,7α,12α,26-tetrol-27-sulfate (5α-cyprinol sulfate)	4.59	1.1	4.36	0.75	None
BI37	5β-Cholestan-3α,7α,12α,26,27-pentol (5α-cyprinol)	None		None		50
BI38	5β-Cholestanic acid-3α,7α,12α-triol	None		None		200
BI39	5α-Cholestan-7α,16α-diol-3β,27-disulfate (5β-myxinol disulfate)	None		None		200
BI40	5α-Cholestan-3β,7α,16α,27-tetrol (5β-myxinol)	None		None		100
BI41	5β-Cholanic acid-3α-sulfate (lithocholic acid sulfate)	3.93	0.69	4.61	0.24	None
BI42	5β-Cholanic acid-3α,12α-diol-7-one (7-ketodeoxycholic acid)	4.24	0.49	None		None
BI43	5β-Cholanic acid-3α-sulfate *N*-(carboxymethyl)-amide (glycolithocholic acid)	4.25	0.19	None		None
BI44	5β-Cholanic acid-3α-sulfate *N*-(2-sulfoethyl)-amide (taurolithocholic acid)	4.08	0.55	None		None
BI45	5α-Cholestan-3α-ol	None		None		None
BI46	5β-Cholanic acid-3α,6β,7β-triol *N*-(2-sulfoethyl)-amide (tauro-β-muricholic acid)	None		None		None
BI47	7α-Hydroxycholesterol	None		None		None
BI48	5β-Cholanic acid-3-one	5.08	0.42	None		None
BI49	5β-Cholanic acid-3α-acetate (lithocholic acid acetate)	5.92	0.54	5.89	0.10	None
BI50	5β-Cholanic acid-3α-acetate methyl ester (lithocholic acid acetate methyl ester)	5.96	0.49	None		None

Activities are in –log(EC_{50}), with EC_{50} in molar units for the activation of human or zebrafish PXR. Efficacy is relative to 10 μM rifampicin (human PXR) or 20 μM 5α-androstan-3α-ol (zebrafish PXR). Toxicity is the lowest concentration in micromolar that produced significant toxicity in the HepG2 cells.
