# Supplementary material for: Evolution of pharmacologic specificity in the pregnane X receptor
Source: BMC Evol Biol. 2008 Apr 2;8:103. doi: 10.1186/1471-2148-8-103 (PMC2358886; doi:10.1186/1471-2148-8-103)
Supplement: Additional file 3 — Comparison of bile salt activation of human and zebrafish PXRs. Comparison of bile salt synthetic pathways for humans and zebrafish, indicating which bile salts and intermediates activate human and zebrafish PXRs. [file 1471-2148-8-103-S3.pdf]

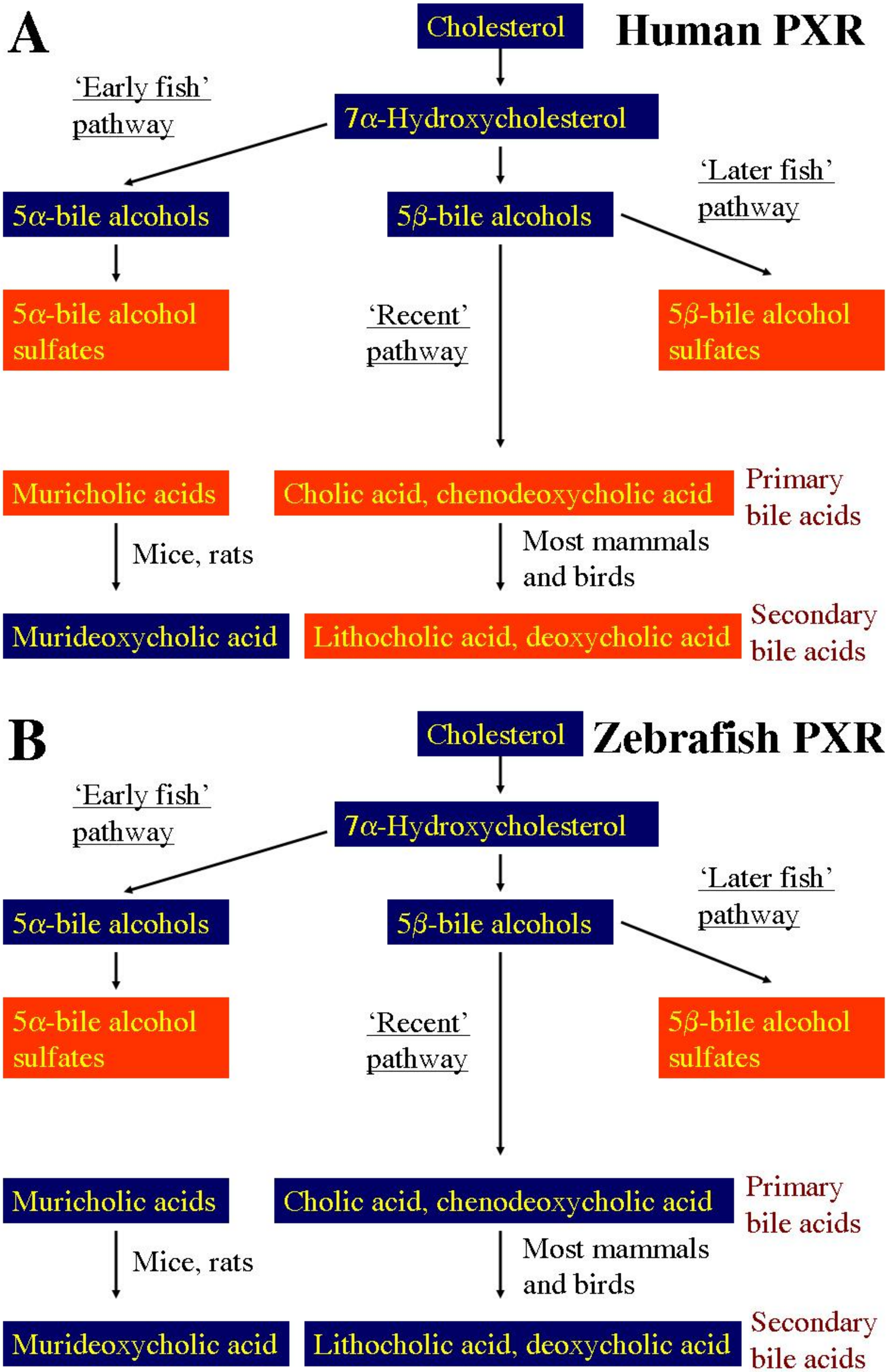
A
Cholesterol
Human PXR
'Early fish' pathway
7α-Hydroxycholesterol
5α-bile alcohols
5β-bile alcohols
'Later fish' pathway
5α-bile alcohol sulfates
'Recent' pathway
5β-bile alcohol sulfates
Muricholic acids
Cholic acid, chenodeoxycholic acid
Primary bile acids
Mice, rats
Most mammals and birds
Murideoxycholic acid
Lithocholic acid, deoxycholic acid
Secondary bile acids
B
Cholesterol
Zebrafish PXR
'Early fish' pathway
7α-Hydroxycholesterol
5α-bile alcohols
5β-bile alcohols
'Later fish' pathway
5α-bile alcohol sulfates
'Recent' pathway
5β-bile alcohol sulfates
Muricholic acids
Cholic acid, chenodeoxycholic acid
Primary bile acids
Mice, rats
Most mammals and birds
Murideoxycholic acid
Lithocholic acid, deoxycholic acid
Secondary bile acids

Additional file 3 - PXR activation and bile salt pathways

All bile salts are derived from cholesterol, a 27-carbon molecule. The earliest bile salt pathway to evolve was to create bile alcohol sulfates with the 5α-orientation (A/B *trans*) [1, 2]. This pathway is used by the hagfish [3] and sea lamprey [4] (two examples from the superclass Agnatha), as well as African and Western clawed frogs (L.R. Hagey and M.D. Krasowski, unpublished data) and zebrafish [5]. The next pathway to evolve was likely 5β-bile alcohol sulfates, a route used in most cartilaginous fish (except Agnatha) but also found in some teleost fish, amphibians, and even a handful of mammals [1, 2, 6]. The evolutionarily most recent bile salt pathways are 24-carbon bile acids. These types of bile acids are produced by most mammals and birds, and some reptiles and teleost fish [1, 2, 6]. Primary bile acids are synthesized in the liver. Secondary bile acids are generated typically by intestinal bacterial enzymatic reactions on primary bile acids. (A) Human PXR is activated by both 'early' and 'recent' bile salts. Bile salts that activate human PXR are indicated in red. (B) Zebrafish PXR is activated only by 'early' bile salts. Bile salts that activate zebrafish PXR are indicated in red.

References:

1. Haslewood GAD: **Bile salt evolution**. *J Lipid Res* 1967, **8**:535-550.
2. Moschetta A, Xu F, Hagey LR, van Berge Henegouwen GP, van Erpecum KJ, Brouwers JF, Cohen JC, Bierman M, Hobbs HH, Steinbach JH *et al*: **A phylogenetic survey of biliary lipids in vertebrates**. *J Lipid Res* 2005, **46**:2221-2232.
3. Haslewood GAD: **Comparative studies of bile salts. Myxinol disulphate, the principal bile salt of hagfish (*Myxinidae*)**. *Biochem J* 1966, **100**:233-237.

4. Haslewood GAD, Tökés L: **Comparative studies of bile salts: bile salts of the lamprey *Petromyzon marinus* L.** *Biochem J* 1969, **114**:179-184.
5. Farber SA, Pack M, Ho S-Y, Johnson DL, Wagner DS, Dosch R, Mullins MC, Hendrickson HS, Hendrickson EK, Halpern ME: **Genetic analysis of digestive physiology using fluorescent phospholipid reporters**. *Science* 2001, **292**:1385-1388.
6. Une M, Hoshita T: **Natural occurrence and chemical synthesis of bile alcohols, higher bile acids, and short side chain bile acids**. *Hiroshima J Med Sci* 1994, **43**:37-67.
