# Supplementary material for: Evolution of pharmacologic specificity in the pregnane X receptor
Source: BMC Evol Biol. 2008 Apr 2;8:103. doi: 10.1186/1471-2148-8-103 (PMC2358886; doi:10.1186/1471-2148-8-103)
Supplement: Additional file 4 — Table summary of statistics of the pharmacophore models. Summary of the statistics for the pharmacophore models of activation of PXRs. [file 1471-2148-8-103-S4.pdf]

Additional file 4: Summary of pharmacophore models

Receptor	R	Total cost –null (difference)	Key features	Summary
Human PXR	0.70	109.54 – 146.08 (36.54)	4 hydrophobic and 4 excluded volumes	Large pharmacophore
Zebrafish PXR	0.78	113.59 – 141.24 (27.65)	1 hydrogen bond acceptor, 3 hydrophobes, 4 excluded volumes	Compact pharmacophore
Mouse PXR	0.78	102.21 – 153.87 (51.66)	4 Hydrophobic and 4 excluded volumes	Large – similar to human PXR
Rat PXR	0.72	96.0 – 124.5 (28.5)	4 Hydrophobic and 4 excluded volumes	Large – similar to human PXR
Rabbit PXR	0.82	91.39 -105.47 (14.08)	1 hydrogen bond acceptor, 3 hydrophobes	Compact pharmacophore
Chicken PXR	0.73	110.8 – 146.21 (35.41)	1 hydrogen bond acceptor, 4 hydrophobes, 4 excluded volumes	Large, possibly symmetrical, pharmacophore
