# Supplementary material for: Evolution of pharmacologic specificity in the pregnane X receptor
Source: BMC Evol Biol. 2008 Apr 2;8:103. doi: 10.1186/1471-2148-8-103 (PMC2358886; doi:10.1186/1471-2148-8-103)
Supplement: Additional file 5 — Additional data for zebrafish PXR, Xenopus tropicalis PXR, and Ciona intestinalis VDR/PXR. Summary of data for screening of compounds as possible activators for zebrafish PXR, Xenopus tropicalis PXR, and the Ciona VDR/PXR. [file 1471-2148-8-103-S5.pdf]

Additional file 5: Additional data for zebrafish PXR, *Xenopus tropicalis* PXR, and *Ciona intestinalis* VDR/PXR

Compound	Classification	Source	Effect on *Xenopus tropicalis* PXR	Effect on zebrafish PXR	Effect on *Ciona intestinalis* VDR/PXR
**25-Hydroxyvitamin D_3**	Vitamin D receptor agonist	BIOMOL (Nuclear receptor ligand library # 1)	No effect	No effect	No effect
**Retinoic acid, all trans**	Retinoid X receptor agonist	BIOMOL (Nuclear receptor ligand library # 2)	No effect	No effect	No effect
**9-*cis* Retinoic acid**	Retinoic acid receptor agonist	BIOMOL (Nuclear receptor ligand library # 3)	No effect	No effect	No effect
**13-*cis* Retinoic acid**	Retinoic acid receptor agonist	BIOMOL (Nuclear receptor ligand library # 4)	No effect	No effect	No effect
**4-Hydroxyphenylretinamide**	Retinoic acid receptor agonist	BIOMOL (Nuclear receptor ligand library # 5)	No effect	No effect	No effect
**AM-580**	Retinoic acid receptor agonist	BIOMOL (Nuclear receptor ligand library # 6)	No effect	No effect	No effect
**TTNPB**	Retinoic acid receptor agonist	BIOMOL (Nuclear receptor ligand library # 7)	No effect	No effect	No effect
**Methoprene acid**	Retinoid X receptor agonist	BIOMOL (Nuclear receptor ligand library # 8)	No effect	No effect	No effect
**WY-14643**	Peroxisome proliferator-activated receptor agonist	BIOMOL (Nuclear receptor ligand library # 9)	No effect	No effect	No effect
**Ciglitazone**	Peroxisome proliferator-activated receptor-gamma agonist	BIOMOL (Nuclear receptor ligand library # 10)	No effect	No effect	No effect
**Tetradecylthioacetic acid**	Peroxisome proliferator-activated receptor agonist	BIOMOL (Nuclear receptor ligand library # 11)	No effect	No effect	No effect
**5,8,11,14-Eicosatetranoic acid**	Peroxisome proliferator-activated receptor-alpha agonist	BIOMOL (Nuclear receptor ligand library # 12)	No effect	No effect	No effect
**6-Formylindolo [3,2-*b*] carbazole**	Aryl hydroxycarbon receptor agonist	BIOMOL (Nuclear receptor ligand library # 13)	No effect	**EC_{50}=1.4, ε=1.28**	**EC_{50}=0.86, ε=1.28**
**Dindoylmethane**	Aryl hydroxycarbon receptor agonist	BIOMOL (Nuclear receptor ligand library # 14)	No effect	No effect	No effect
**Acetyl-farnesyl-cysteine**	Farnesoid	BIOMOL (Nuclear receptor ligand library # 15)	No effect	No effect	No effect
**S-Farnesyl-L-cysteine methyl ester**	Farnesoid	BIOMOL (Nuclear receptor ligand library # 16)	No effect	No effect	No effect
**Acetyl-geranylgeranyl-cysteine**	Farnesoid	BIOMOL (Nuclear receptor ligand library # 17)	No effect	No effect	No effect
**Acetyl-geranyl-cysteine**	Farnesoid	BIOMOL (Nuclear receptor ligand library # 18)	No effect	No effect	No effect
**Farnesylthioacetic acid**	Farnesoid	BIOMOL (Nuclear receptor ligand library # 19)	No effect	No effect	No effect
**Bezafibrate**	Peroxisome proliferator-activated receptor-alpha agonist	BIOMOL (Nuclear receptor ligand library # 20)	No effect	No effect	No effect
**LY 171883**	Peroxisome proliferator-activated receptor-gamma agonist	BIOMOL (Nuclear receptor ligand library # 21)	No effect	No effect	No effect
**15-Deoxy-D12,14-prostaglandin J2**	Peroxisome proliferator-activated receptor-gamma agonist	BIOMOL (Nuclear receptor ligand library # 22)	No effect	No effect	No effect
**Troglitazone**	Peroxisome proliferator-activated receptor-gamma agonist	BIOMOL (Nuclear receptor ligand library # 23)	No effect	No effect	No effect
**CITCO**	Constitutive androstane receptor agonist	BIOMOL (Nuclear receptor ligand library # 24)	No effect	No effect	No effect
**Paxilline**	Liver X receptor agonist	BIOMOL (Nuclear receptor ligand library # 25)	No effect	No effect	No effect
**24(S)-Hydroxycholesterol**	Liver X receptor agonist	BIOMOL (Nuclear receptor ligand library # 26)	No effect	No effect	No effect
**24(S)-,25-Epoxycholesterol**	Liver X receptor agonist	BIOMOL (Nuclear receptor ligand library # 27)	No effect	No effect	No effect
**Pregnenolone-16α-carbonitrile**	Pregnane X receptor agonist	BIOMOL (Nuclear receptor ligand library # 28)	No effect	No effect	No effect
**Carbacyclin**	Peroxisome proliferator-activated receptor-delta agonist	BIOMOL (Nuclear receptor ligand library # 29)	No effect	No effect	No effect
**Clofibric acid**	Peroxisome proliferator-activated receptor-alpha agonist	BIOMOL (Nuclear receptor ligand library # 30)	No effect	No effect	No effect
**BADGE**	Peroxisome proliferator-activated receptor-gamma agonist	BIOMOL (Nuclear receptor ligand library # 31)	No effect	No effect	No effect
**GW 9662**	Peroxisome proliferator-activated receptor-gamma agonist	BIOMOL (Nuclear receptor ligand library # 32)	No effect	No effect	No effect
**Gemfibrozil**	Peroxisome proliferator-activated receptor-alpha agonist	BIOMOL (Nuclear receptor ligand library # 33)	No effect	No effect	No effect
**GW 7647**	Peroxisome proliferator-activated receptor-alpha agonist	BIOMOL (Nuclear receptor ligand library # 34)	No effect	No effect	No effect
**3,5-Diiodo-L-thyronine**	Thyroid hormone receptor agonist	BIOMOL (Nuclear receptor ligand library # 35)	No effect	No effect	No effect
**3,5-Diiodo-L-tyroside dihydrate**	Thyroid hormone receptor agonist	BIOMOL (Nuclear receptor ligand library # 36)	No effect	No effect	No effect
**all-trans-Retinol**	Retinoid	BIOMOL (Nuclear receptor ligand library # 37)	No effect	No effect	No effect
**13-cis-Retinol**	Retinoid	BIOMOL (Nuclear receptor ligand library # 38)	No effect	No effect	No effect
**Retinyl acetate**	Retinoid	BIOMOL (Nuclear receptor ligand library # 39)	No effect	No effect	No effect
**3,5-Diiodo-4-hydroxyphenylpropionic acid**	Thyroid hormone receptor agonist	BIOMOL (Nuclear receptor ligand library # 40)	No effect	No effect	No effect
**Cholic acid**	Bile acid	BIOMOL (Nuclear receptor ligand library # 41)	No effect	No effect	No effect
**Deoxycholic acid**	Bile acid	BIOMOL (Nuclear receptor ligand library # 42)	No effect	No effect	No effect
**Chenodeoxycholic acid**	Bile acid	BIOMOL (Nuclear receptor ligand library # 43)	No effect	No effect	No effect
**Glycocholic acid hydrate**	Bile acid	BIOMOL (Nuclear receptor ligand library # 44)	No effect	No effect	No effect
**Glycodeoxycholic acid monohydrate**	Bile acid	BIOMOL (Nuclear receptor ligand library # 45)	No effect	No effect	No effect
**Taurocholic acid sodium salt**	Bile acid	BIOMOL (Nuclear receptor ligand library # 46)	No effect	No effect	No effect
**Taurodeoxycholic acid sodium salt**	Bile acid	BIOMOL (Nuclear receptor ligand library # 47)	No effect	No effect	No effect
**Rifampicin**	Pregnane X receptor agonist	BIOMOL (Nuclear receptor ligand library # 48)	No effect	No effect	No effect
**Dexamethasone**	Pregnane steroid	BIOMOL (Nuclear receptor ligand library # 49)	No effect	No effect	No effect
**Lithocholic acid**	Bile acid	BIOMOL (Nuclear receptor ligand library # 50)	No effect	No effect	No effect
**5β-Pregnan-3,20-dione**	Pregnane steroid	BIOMOL (Nuclear receptor ligand library # 51)	No effect	**EC_{50}=0.83, ε=0.85**	No effect
**Hyperforin**	Pregnane X receptor agonist	BIOMOL (Nuclear receptor ligand library # 52)	No effect	No effect	No effect
**Farnesol**	Farnesoid	BIOMOL (Nuclear receptor ligand library # 53)	No effect	**EC_{50}=20.6, ε=0.43**	No effect
**3α,5α-Androstenol**	Androstane steroid	BIOMOL (Nuclear receptor ligand library # 54)	No effect	**EC_{50}=3.6, ε=1.02**	No effect

**Compound**	**Classification**	**Source**	**Effect on *Xenopus tropicalis* PXR**	**Effect on zebrafish PXR**	**Effect on *Ciona intestinalis* VDR/PXR**
**3α,5α-Androstanol**	Androstane steroid	BIOMOL (Nuclear receptor ligand library # 55)	No effect	**EC_{50}=4.6, ε=1.0**	No effect
**Z-Guggulsterone**	Farnesoid X receptor antagonist	BIOMOL (Nuclear receptor ligand library # 56)	No effect	No effect	No effect
**TCPOBOP**	Constitutive androstane receptor agonist	BIOMOL (Nuclear receptor ligand library # 57)	No effect	No effect	No effect
**N-Oleoylethanolamide**	Peroxisome proliferator-activated receptor-alpha agonist	BIOMOL (Nuclear receptor ligand library # 58)	No effect	No effect	No effect
**LY 295427**	Low density lipoprotein receptor up-regulator	BIOMOL (Nuclear receptor ligand library # 59)	No effect	No effect	No effect
**Geranygeraniol**	Farnesoid	BIOMOL (Nuclear receptor ligand library # 60)	No effect	No effect	No effect
**6α-Fluorotestosterone**	Androstane steroid	BIOMOL (Nuclear receptor ligand library # 61)	No effect	No effect	No effect
**Tamoxifen**	Estrogen receptor antagonist	BIOMOL (Nuclear receptor ligand library # 62)	No effect	No effect	No effect
**Mifepristone**	Progesterone receptor antagonist	BIOMOL (Nuclear receptor ligand library # 63)	No effect	No effect	No effect
**Estrone**	Estrane steroid	BIOMOL (Nuclear receptor ligand library # 64)	No effect	No effect	No effect
**13(S)-Hydroxy-9Z,11E-octadecadienoic acid**	Peroxisome proliferator-activated receptor-gamma agonist	BIOMOL (Nuclear receptor ligand library # 65)	No effect	No effect	No effect
**Cortisone**	Pregnane steroid	BIOMOL (Nuclear receptor ligand library # 66)	No effect	No effect	No effect
**Progesterone**	Pregnane steroid	BIOMOL (Nuclear receptor ligand library # 67)	No effect	No effect	No effect
**17β-Estradiol**	Estrane steroid	BIOMOL (Nuclear receptor ligand library # 68)	No effect	No effect	No effect
**Pregnenolone**	Pregnane steroid	BIOMOL (Nuclear receptor ligand library # 69)	No effect	**EC_{50}=0.48, ε=2.05**	No effect
**Androstenedione**	Androstane steroid	BIOMOL (Nuclear receptor ligand library # 70)	No effect	**EC_{50}=3.6, ε=0.14**	No effect
**1α,-25-Dihydroxyvitamin D_3**	Vitamin D receptor agonist	BIOMOL (Nuclear receptor ligand library # 71)	No effect	No effect	No effect
**Docosa-4Z,7Z,10Z,13Z,16Z,19Z-hexanoic acid**	Retinoid X receptor agonist	BIOMOL (Nuclear receptor ligand library # 72)	No effect	No effect	No effect
**3-Methylcholanthrene**	Aryl hydroxycarbon receptor agonist	BIOMOL (Nuclear receptor ligand library # 73)	No effect	No effect	No effect
**Aciretin**	Retinoid	BIOMOL (Nuclear receptor ligand library # 74)	No effect	No effect	No effect
**Pioglitazone HCl**	Peroxisome proliferator-activated receptor-gamma agonist	BIOMOL (Nuclear receptor ligand library # 75)	No effect	No effect	No effect
**4-Hydroxretinoic acid**	Retinoid	BIOMOL (Nuclear receptor ligand library # 76)	No effect	No effect	No effect
**12-Ketolithocholic acid**	Bile acid	Steraloids	No effect	No effect	No effect
**16,(5α)-Androsten-3β-ol**	Androstane steroid	Steraloids	No effect	No effect	No effect
**16,(5α)-Androsten-3-one**	Androstane steroid	Steraloids	No effect	No effect	No effect
**16α-Hydroxyestrone**	Estrane steroid	Steraloids	No effect	No effect	No effect
**17-Hydroxypregnenolone**	Pregnane steroid	Steraloids	No effect	No effect	No effect
**17-Hydroxyprogesterone**	Pregnane steroid	Steraloids	No effect	No effect	No effect
**23-Nordeoxycholic acid**	Bile acid	Steraloids	No effect	No effect	No effect
**2-Hydroxyestrone**	Estrane steroid	Steraloids	No effect	No effect	No effect
**4,16-Androstadien-3-one**	Androstane steroid	Steraloids	No effect	No effect	No effect
**4-Androsten-17α-3-one glucosiduronate**	Androstane steroid	Steraloids	No effect	No effect	No effect
**4-Androsten-17α-ol-3-one sulfate**	Androstane steroid	Steraloids	No effect	No effect	No effect
**4-Methoxyestrone**	Estrane steroid	Steraloids	No effect	No effect	No effect
**4-Pregnen-17,20β-diol-3-one sulfate**	Pregnane steroid	Steraloids	No effect	No effect	No effect
**5,16-Androstadien-3β-ol**	Androstane steroid	Steraloids	No effect	No effect	No effect
**5α-Androstan-3,17-dione (androstanedione)**	Androstane steroid	Steraloids	No effect	No effect	No effect
**5α-Androstan-3α,17β-diol (dihydroandrosterone)**	Androstane steroid	Steraloids	No effect	No effect	No effect
**5α-Androstan-3β-ol**	Androstane steroid	Steraloids	No effect	No effect	No effect
**5α-Cholestan-3α,7α,16α,27-tetrol (5α-myxinol)**	Bile alcohol	Lee Hagey	No effect	No effect	No effect
**5α-Pregnan-3α,11β,21-triol-20-one**	Pregnane steroid	Steraloids	No effect	No effect	No effect
**5β-Androstan-3α,11β-17-one**	Androstane steroid	Steraloids	No effect	No effect	No effect
**5β-Androstan-3α-ol-11,17-one**	Pregnane steroid	Steraloids	No effect	No effect	No effect
**5β-Androstan-3β-ol**	Androstane steroid	Steraloids	No effect	No effect	No effect
**5β-Cholan-3α,7α,12α,24-tetrol**	Bile alcohol	Steraloids	No effect	No effect	No effect
**5β-Cholan-3α,7α,12α-triol**	Bile salt intermediate	Steraloids	No effect	No effect	No effect
**5β-Cholestan-3α,7α,12α,24,26,27-tetrol (5β-scymnol)**	Bile alcohol	Lee Hagey	No effect	No effect	No effect
**5β-Pregnan-3α,20α-diol**	Pregnane steroid	Steraloids	No effect	No effect	No effect
**5β-Pregnan-3α,20β,diol**	Pregnane steroid	Steraloids	No effect	No effect	No effect
**5β-Pregnan-3α-ol-20-one**	Pregnane steroid	Steraloids	No effect	No effect	No effect
**5β-scymnol 27-sulfate**	Bile alcohol	Lee Hagey	No effect	No effect	No effect
**5-Pregnen-3β,17-diol 3-sulfate**	Pregnane steroid	Steraloids	No effect	No effect	No effect
**7,12-Diketolithocholic acid**	Bile acid	Steraloids	No effect	No effect	No effect
**7-Ketodeoxycholic acid**	Bile acid	Steraloids	No effect	No effect	No effect
**7-Ketolithocholic acid**	Bile acid	Steraloids	No effect	No effect	No effect
**Acetaminophen**	Xenobiotic	Sigma	No effect	No effect	No effect
**Aldosterone**	Pregnane steroid	Steraloids	No effect	No effect	No effect

Compound	Classification	Source	Effect on *Xenopus tropicalis* PXR	Effect on zebrafish PXR	Effect on *Ciona intestinalis* VDR/PXR
**Allocholic acid**	Bile acid	Toronto Research Chemicals	No effect	No effect	No effect
**Allopregnanolone (5α-pregnan-3α,20α-diol)**	Pregnane steroid	Steraloids	No effect	No effect	No effect
**Allopregnanolone (5α-pregnan-3α-ol-20-one)**	Pregnane steroid	Steraloids	No effect	No effect	No effect
**Alpha-tocopherol**	Vitamin E derivative	EMD	No effect	No effect	No effect
**α-Muricholic acid**	Bile acid	Steraloids	No effect	No effect	No effect
**Androsterone**	Androstane steroid	Steraloids	No effect	No effect	No effect
**Beta-carotene**	Vitamin	EMD	No effect	No effect	No effect
**Beta-sitosterol**	Cholesterol derivative	Steraloids	No effect	No effect	No effect
**Beta-tocopherol**	Vitamin E derivative	EMD	No effect	No effect	No effect
**β-Muricholic acid**	Bile acid	Steraloids	No effect	No effect	No effect
**Caffeine**	Xenobiotic	Sigma	No effect	No effect	No effect
**Campesterol**	Cholesterol derivative	Steraloids	No effect	No effect	No effect
**Carbamazepine**	Xenobiotic	Sigma	No effect	No effect	**EC_{50} > 10, ε~0.10**
**Chlorzoxazone**	Xenobiotic	Sigma	No effect	No effect	No effect
**Cholestanol sulfate (5α-cholestan-3β-ol sulfate)**	Cholesterol derivative	Steraloids	No effect	No effect	No effect
**Cholesteryl sulfate (5-cholesten-3β-ol)**	Cholesterol derivative	Steraloids	No effect	No effect	No effect
**Clotrimazole**	Xenobiotic	Sigma	No effect	No effect	No effect
**Cortexolone (4-Pregnen-17,21-diol-3,20-dione)**	Pregnane steroid	Steraloids	No effect	No effect	No effect
**Cortexone (4-Pregnen-21-ol-3,20-dione)**	Pregnane steroid	Steraloids	No effect	No effect	No effect
**Corticosterone**	Pregnane steroid	Steraloids	No effect	No effect	No effect
**Cortisol**	Pregnane steroid	Steraloids	No effect	No effect	No effect
**Cortol**	Pregnane steroid	Steraloids	No effect	No effect	No effect
**Cortolone**	Pregnane steroid	Steraloids	No effect	No effect	No effect
**Cycloartenol**	Plant steroid	Steraloids	No effect	No effect	No effect
**Cyclosporine**	Xenobiotic	Sigma	No effect	No effect	No effect
**DHEA**	Androstane steroid	Steraloids	No effect	No effect	No effect
**DHEA sulfate**	Androstane steroid	Steraloids	No effect	No effect	No effect
**Dihydrotestosterone**	Androstane steroid	Steraloids	No effect	No effect	No effect
**Ecdysone**	Ecdysone receptor agonist	Steraloids	No effect	No effect	No effect
**Epiandrosterone (5α-androstan-3β-ol-17-one)**	Pregnane steroid	Steraloids	No effect	No effect	No effect
**Epicoprostanol sulfate (5β-cholestan-3α-ol sulfate)**	Cholesterol derivative	Steraloids	No effect	No effect	No effect
**Epitestosterone**	Androstane steroid	Steraloids	No effect	No effect	No effect
**Epitestosterone 17-glucuronide**	Androstane steroid	Steraloids	No effect	No effect	No effect
**Epitestosterone 17-sulfate**	Androstane steroid	Steraloids	No effect	No effect	No effect
**Estetrol**	Estrane steroid	Steraloids	No effect	No effect	No effect
**Estradiol 3-glucuronide**	Estrane steroid	Steraloids	No effect	No effect	No effect
**Estradiol 3-sulfate**	Estrane steroid	Steraloids	No effect	No effect	No effect
**Estriol**	Estrane steroid	Steraloids	No effect	No effect	No effect
**Estrone 3-sulfate**	Estrane steroid	Steraloids	No effect	No effect	No effect
**Ethinyl estradiol**	Estrane steroid (synthetic)	Steraloids	No effect	No effect	No effect
**Etiocholanolone**	Androstane steroid	Steraloids	No effect	No effect	No effect
**Flurbiprofen**	Xenobiotic	Sigma	No effect	No effect	No effect
**Glycochenodeoxycholic acid**	Bile acid	Sigma	No effect	No effect	No effect
**Glycodeoxycholic acid**	Bile acid	Steraloids	No effect	No effect	No effect
**GW3965**	Liver X receptor agonist	Sigma	No effect	No effect	No effect
**Hyodeoxycholic acid (5β-Cholanic acid-3α,6α-diol)**	Bile acid	Steraloids	No effect	No effect	No effect
**Ketoconazole**	Xenobiotic	Sigma	No effect	No effect	No effect
**Lanosterol**	Cholesterol derivative	Steraloids	No effect	No effect	No effect
**Lathosterol**	Cholesterol derivative	Steraloids	No effect	No effect	No effect
**Levonorgestrol**	Progesterone receptor agonist	Steraloids	No effect	No effect	No effect
**Murocholic acid (5β-Cholanic acid-3α,6β-diol)**	Bile acid	Steraloids	No effect	No effect	No effect
**Mycophenolic acid**	Xenobiotic	Sigma	No effect	No effect	No effect
**n-Butyl 4-aminobenzoate**	Pregnane X receptor agonist (Xenopus laevis)	Sigma	**EC_{50}=14.4, ε=0.89**	**EC_{50}=13.9, ε=0.69**	**EC_{50}=16.5, ε=0.54**
**Nifedipine**	Xenobiotic	Sigma	No effect	No effect	No effect
**Norethindrone**	Progesterone receptor agonist	Steraloids	No effect	No effect	No effect
**n-Propyl 4-hydroxybenzoate**	Pregnane X receptor agonist (Xenopus laevis)	Sigma	**EC_{50}=34.9, ε=1.0**	**EC_{50}=48.6, ε=0.54**	No effect
**Paclitaxel**	Xenobiotic	Sigma	No effect	No effect	No effect

Compound	Classification	Source	Effect on *Xenopus tropicalis* PXR	Effect on zebrafish PXR	Effect on *Ciona intestinalis* VDR/PXR
**Phenobarbital**	Constitutive androstane receptor agonist	Sigma	No effect	No effect	No effect
**Pregnanediol glucuronide**	Pregnane steroid	Steraloids	No effect	No effect	No effect
**Pregnanolone sulfate (5β-pregnan-3α-ol-20-one sulfate)**	Pregnane steroid	Steraloids	No effect	No effect	No effect
**Pregnenolone sulfate**	Pregnane steroid	Steraloids	No effect	No effect	No effect
**Reserpine**	Xenobiotic	Sigma	No effect	No effect	No effect
**Retinol**	Vitamin A derivative	Sigma	No effect	No effect	No effect
**SR12813**	Pregnane X receptor agonist	Sigma	No effect	No effect	No effect
**Taurochenodeoxycholic acid**	Bile acid	Sigma	No effect	No effect	No effect
**Taurohyodeoxycholic acid**	Bile acid	Steraloids	No effect	No effect	No effect
**Testosterone**	Androstane steroid	Steraloids	No effect	No effect	No effect
**Tetrahydrocortisol**	Pregnane steroid	Steraloids	No effect	No effect	No effect
**Tetrahydrocortisone**	Pregnane steroid	Steraloids	No effect	No effect	No effect
**Vitamin K2**	Vitamin K derivative	Sigma	No effect	No effect	No effect
**Vitamin K3**	Vitamin K derivative	Sigma	No effect	No effect	No effect
**ω-Muricholic acid**	Bile acid	Steraloids	No effect	No effect	No effect
