# Supplementary material for: Evolution of pharmacologic specificity in the pregnane X receptor
Source: BMC Evol Biol. 2008 Apr 2;8:103. doi: 10.1186/1471-2148-8-103 (PMC2358886; doi:10.1186/1471-2148-8-103)
Supplement: Additional file 6 — HIPHOP model for Ciona VDR/PXR. HIPHOP alignment of carbamazepine, 6-formylindolo-[3,2-b]carbazole, and n-butyl-p-aminobenzoate as activators of Ciona VDR/PXR. [file 1471-2148-8-103-S6.pdf]

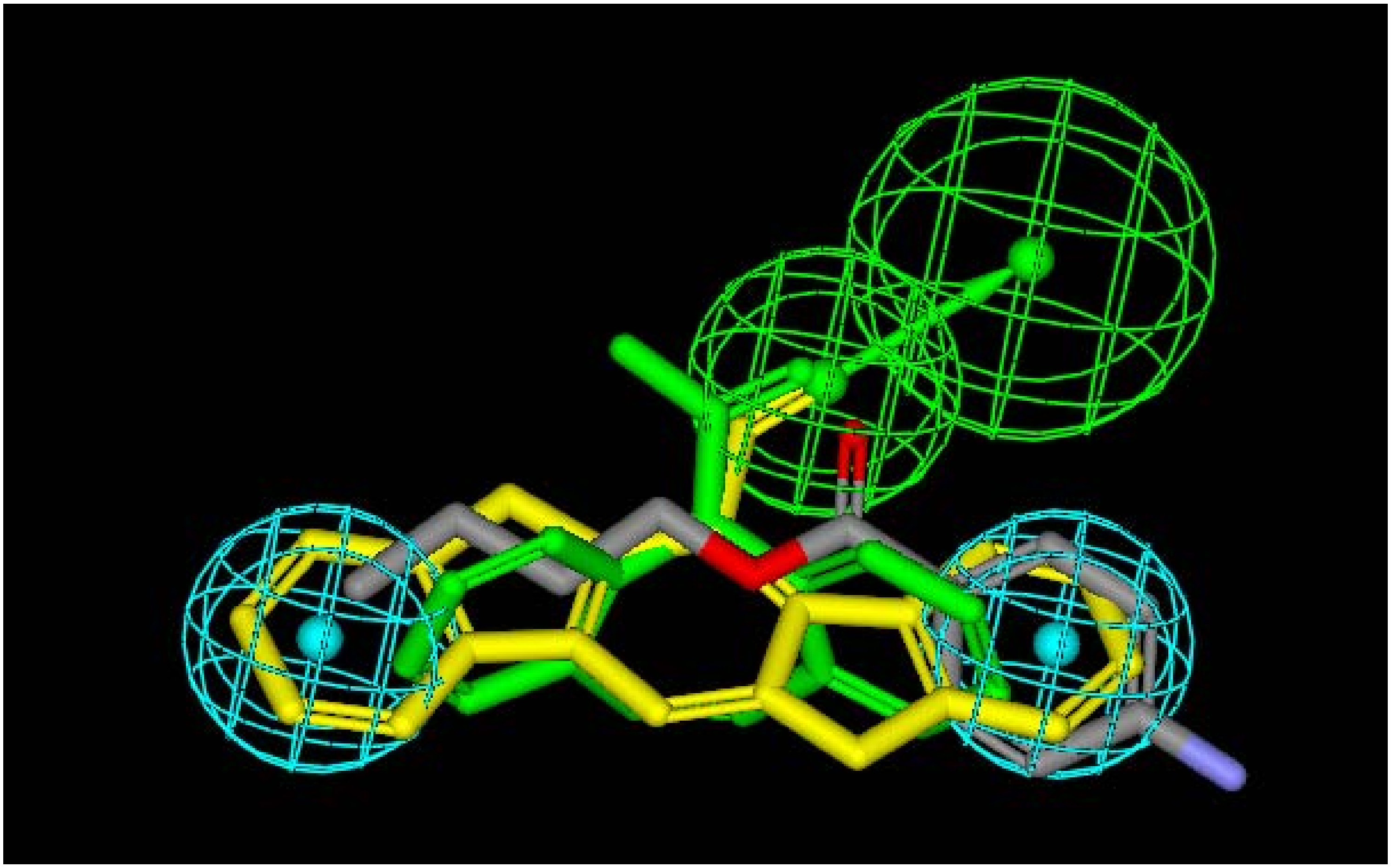

Additional file 6 – HIPHOP alignment of carbamazepine (green), 6-formylindolo-[3,2-b]carbazole (yellow), and *n*-butyl-*p*-aminobenzoate (grey) that activate *Ciona* VDR/PXR. Green spheres indicate hydrogen bond acceptor and vector. Cyan spheres indicate hydrophobic.
