# Supplementary material for: Evolution of pharmacologic specificity in the pregnane X receptor
Source: BMC Evol Biol. 2008 Apr 2;8:103. doi: 10.1186/1471-2148-8-103 (PMC2358886; doi:10.1186/1471-2148-8-103)
Supplement: Additional file 7 — Reconstructed ancestral sequences. Detailed data for the reconstruction of ancestral sequences. [file 1471-2148-8-103-S7.pdf]

Additional file 7: Ancestral reconstruction
See Methods for details.

Domain (DBD=DNA-binding domain LBD=Ligand-binding domain H=helix)	Site #	Human PXR amino acid #	VDR Ligand-binding residue?	PXR Ligand-binding residue?	CAR Ligand-binding residue?	AncR1 Prior to VDR/PXR split	Posterior probability (PP)	AncR2 Prior to mammalian/fish PXR split	Posterior probability (PP)	AncR3 Prior to mammalian PXR/ CXR-CAR split	Posterior probability (PP)
DBD	1	78				K	0.964	R	0.491	K	0.98
DBD	2	79				I	0.619	A	0.905	V	0.531
DBD	3	80				C	1	C	1	C	1
DBD	4	81				G	0.964	G	1	G	0.563
DBD	5	82				V	0.988	V	0.998	V	0.996
DBD	6	83				C	1	C	1	C	1
DBD	7	84				G	0.999	G	1	G	1
DBD	8	85				D	0.997	D	0.999	D	0.999
DBD	9	86				K	0.532	Q	0.996	R	0.763
DBD	10	87				A	0.997	A	0.999	A	0.999
DBD	11	88				T	0.988	K	0.817	T	0.998
DBD	12	89				G	1	G	1	G	1
DBD	13	90				Y	0.991	Y	1	Y	1
DBD	14	91				H	1	H	1	H	1
DBD	15	92				F	0.995	F	1	F	1
DBD	16	93				N	0.999	N	1	N	0.974
DBD	17	94				A	0.997	A	1	A	0.649
DBD	18	95				M	0.98	W	1	M	0.999
DBD	19	96				T	0.996	T	1	T	0.994
DBD	20	97				C	1	C	1	C	1
DBD	21	98				E	0.997	E	0.999	E	0.999
DBD	22	99				G	1	G	1	G	1
DBD	23	100				C	1	C	1	C	1
DBD	24	101				K	0.998	K	1	K	0.999
DBD	25	102				G	1	G	1	G	1
DBD	26	103				F	1	F	1	F	1
DBD	27	104				F	1	F	1	F	1
DBD	28	105				R	0.998	R	1	R	0.999
DBD	29	106				R	0.998	R	1	R	0.999
DBD	30	107				S	0.874	A	0.999	A	0.933
DBD	31	108				M	0.979	I	0.995	M	0.97
DBD	32	109				K	0.996	K	1	K	0.986
DBD	33	110				R	0.953	R	1	R	0.953
DBD	34	111				N	0.805	T	0.983	N	0.793
DBD	35	112				A	0.96	A	0.444	A	0.639
DBD	36	113				S	0.471	Q	0.974	R	0.55
DBD	37	114				F	0.96	L	1	L	0.986
DBD	38	115				T	0.835	P	0.382	T	0.564
DBD	39	116				C	1	C	1	C	1
DBD	40	117				P	0.985	P	0.981	P	1
DBD	41	118				F	0.974	F	1	F	1
DBD	42	119				N	0.647	R	0.211	R	0.445
DBD	43	120				Q	0.593	L	1	Q	0.61
DBD	44	121				G	0.879	N	0.999	G	0.806
DBD	45	122				N	0.508	K	0.99	S	0.852
DBD	46	123				C	1	C	1	C	1
DBD	47	124				T	0.329	S	0.979	E	0.657
DBD	48	125				I	0.984	I	0.999	I	0.991
DBD	49	126				T	0.962	T	1	T	0.995
DBD	50	127				K	0.996	K	1	K	0.979
DBD	51	128				S	0.578	K	0.99	S	0.681
DBD	52	129				N	0.995	N	1	N	0.77

Domain (DBD=DNA-binding domain LBD=Ligand-binding domain H=helix)	Site #	Human PXR amino acid #	VDR Ligand-binding residue?	PXR Ligand-binding residue?	CAR Ligand-binding residue?	AncR1 Prior to VDR/PXR split	Posterior probability (PP)	AncR2 Prior to mammalian/fish PXR split	Posterior probability (PP)	AncR3 Prior to mammalian PXR/ CXR-CAR split	Posterior probability (PP)
DBD	53	130				R	0.998	R	1	R	0.999
DBD	54	131				R	0.998	R	1	R	0.999
DBD	55	132				H	0.719	Q	0.999	Q	0.894
DBD	56	133				C	1	C	1	C	1
DBD	57	134				Q	1	Q	1	Q	1
DBD	58	135				A	0.993	A	0.971	A	0.999
DBD	59	136				C	1	C	1	C	1
DBD	60	137				R	0.998	R	1	R	0.999
DBD	61	138				L	0.999	L	0.999	L	1
DBD	62	139				K	0.808	R	0.986	Q	0.716
DBD	63	140				K	0.834	K	1	K	0.999
DBD	64	141				C	1	C	1	C	1
DBD	65	142				L	0.378	Q	1	L	1
DBD	66	143				D	0.936	A	0.998	D	0.973
DBD	67	144				I	0.976	I	0.999	I	0.685
DBD	68	145				G	1	G	1	G	1
DBD	69	146				M	1	M	1	M	1
LBD	70	141				P	0.409	V	0.826	A	0.464
LBD	71	142				R	0.409	Q	0.893	G	0.604
LBD	72	143				L	0.993	L	1	L	1
LBD	73	144				S	0.557	S	0.995	T	0.963
LBD-H1	74	145				P	0.497	S	0.98	E	0.501
LBD-H1	75	146				E	0.984	Q	1	E	0.996
LBD-H1	76	147				Q	0.999	Q	1	Q	1
LBD-H1	77	148				Q	0.913	E	0.999	Q	0.98
LBD-H1	78	149				Q	0.724	E	0.986	Q	0.892
LBD-H1	79	150				L	0.72	T	0.987	L	0.742
LBD-H1	80	151				I	0.98	I	0.995	I	0.984
LBD-H1	81	152				A	0.229	R	0.986	Q	0.783
LBD-H1	82	153				T	0.61	E	0.999	E	0.983
LBD-H1	83	154				L	0.999	L	1	L	1
LBD-H1	84	155				V	0.822	V	0.869	V	0.481
LBD-H1	85	156				E	0.416	Y	0.538	D	0.415
LBD-H1	86	157				A	0.966	G	0.789	A	0.999
LBD-H1	87	158				H	0.998	H	1	H	0.988
LBD-H1	88	159				R	0.735	R	0.998	K	0.581
LBD-H1	89	160				K	0.985	K	0.999	K	0.953
LBD-H1	90	161				T	0.978	T	1	T	0.998
LBD	91	162	Yes	No	No	Y	0.688	F	1	F	1
LBD	92	163				D	0.971	D	0.999	D	0.998
LBD	93	164				T	0.629	P	0.94	T	0.665
LBD	94	165				S	0.615	A	0.795	T	0.754
LBD	95	166				Y	0.75	F	1	F	0.999
LBD	96	167				S	0.965	Y	0.997	S	0.993
LBD	97	168				D	0.897	R	0.999	H	0.92
LBD	98	169	Yes	No	Yes	F	0.987	F	1	F	1
LBD	99	170				K	0.819	T	0.76	K	0.914
LBD	100	171				K	0.394	S	0.772	N	0.586
LBD	101	172				F	0.989	F	1	F	0.997
LBD	102	173				R	0.976	R	0.943	R	0.997
LBD	103	174				P	0.999	R	0.396	P	0.995
LBD	104	175				P	0.918	P	0.852	P	0.986
LBD	105	176				K	0.85	K	0.489	E	0.698
LBD	106	177	Yes	No	No	R	0.969	S	0.509	R	0.98
LBD	107	178				G	0.72	T	0.438	L	0.88
LBD	108	179				D	0.351	T	0.407	S	0.891

Domain (DBD=DNA-binding domain LBD=Ligand-binding domain H=helix)	Site #	Human PXR amino acid #	VDR Ligand-binding residue?	PXR Ligand-binding residue?	CAR Ligand-binding residue?	AncR1 Prior to VDR/PXR split	Posterior probability (PP)	AncR2 Prior to mammalian/fish PXR split	Posterior probability (PP)	AncR3 Prior to mammalian PXR/ CXR-CAR split	Posterior probability (PP)
LBD	109	180				S	0.549	L	0.767	S	0.701
LBD	110	181				S	0.894	F	0.997	S	0.628
LBD	111	182				T	0.732	D	0.484	T	0.316
LBD	112	183				Q	0.817	S	0.411	Q	0.95
LBD	113	184				D	0.416	G	0.71	I	0.685
LBD	114	185				P	0.408	H	0.222	P	0.987
LBD	115	186				S	0.457	S	0.995	S	0.298
LBD	116	187				A	0.441	Q	0.441	P	0.584
LBD	117	188				T	0.78	P	0.56	L	0.465
LBD	118	189				S	0.429	M	0.753	S	0.417
LBD	119	190				S	0.841	D	0.299	S	0.641
LBD	120	191				S	0.838	R	0.518	S	0.843
LBD	121					L	0.444	L	0.193	S	0.377
LBD	122	192				S	0.789	N	0.985	S	0.95
LBD	123	193				S	0.425	I	0.982	R	0.366
LBD	124	194				P	0.628	F	0.996	G	0.248
LBD	125	195				D	0.518	A	0.523	E	0.523
LBD	126	196				N	0.617	V	0.985	N	0.532
LBD	127					P	0.564	P	0.25	S	0.514
LBD	128	197				S	0.437	G	0.867	A	0.64
LBD	129	198				E	0.325	D	0.511	P	0.41
LBD	130	199				M	0.335	S	0.988	S	0.348
LBD	131	200				S	0.624	S	0.983	A	0.502
LBD	132	201				S	0.456	P	0.485	S	0.475
LBD	133	202				N	0.346	S	0.984	I	0.418
LBD	134	203				S	0.49	G	0.996	S	0.615
LBD	135	204				S	0.654	P	0.438	S	0.427
LBD	136	205				S	0.412	A	0.99	Q	0.778
LBD	137	206	No	Yes	No	S	0.605	S	0.842	L	0.915
LBD	138	207				C	0.516	S	0.992	C	0.537
LBD	139	208	No	Yes	No	S	0.514	A	0.758	S	0.764
LBD	140	209	No	Yes	No	S	0.527	V	0.987	M	0.433
LBD	141	210				T	0.355	A	0.423	A	0.178
LBD	142	211	No	Yes	No	T	0.307	P	0.328	T	0.432
LBD	143	212				S	0.711	S	0.907	S	0.943
LBD	144	213				S	0.367	P	0.639	L	0.936
LBD	145	214				S	0.376	L	0.996	L	0.5
LBD	146	215				S	0.881	S	0.999	S	0.64
LBD	147					S	0.364	S	0.154	S	0.226
LBD	148	216				S	0.393	S	0.353	S	0.438
LBD	149	217				S	0.738	S	0.999	S	0.638
LBD	150	218				S	0.366	S	0.466	S	0.378
LBD	151	219				M	0.316	S	0.968	S	0.441
LBD	152	220				F	0.433	L	0.519	Y	0.319
LBD	153	221				Q	0.84	P	0.492	Q	0.486
LBD	154	222				S	0.267	P	0.512	V	0.291
LBD	155	223				L	0.473	R	0.996	W	0.429
LBD	156	224				D	0.613	G	0.424	D	0.472
LBD	157	225				S	0.464	E	0.982	N	0.333
LBD	158	226				S	0.315	T	0.991	K	0.747
LBD	159	227				P	0.947	Q	0.798	P	0.924
LBD	160	228				K	0.617	Q	0.994	K	0.374
LBD	161					S	0.392	T	0.522	A	0.279
LBD	162					S	0.573	Q	0.706	Q	0.183
LBD	163					E	0.865	E	0.962	E	0.813
LBD	164					G	0.508	G	0.998	G	0.591

Domain (DBD=DNA-binding domain LBD=Ligand-binding domain H=helix)	Site #	Human PXR amino acid #	VDR Ligand-binding residue?	PXR Ligand-binding residue?	CAR Ligand-binding residue?	AncR1 Prior to VDR/PXR split	Posterior probability (PP)	AncR2 Prior to mammalian/fish PXR split	Posterior probability (PP)	AncR3 Prior to mammalian PXR/ CXR-CAR split	Posterior probability (PP)
LBD	165	228				E	0.718	E	0.997	E	0.687
LBD	166	229				A	0.546	N	0.326	A	0.623
LBD	167	230				E	0.438	A	0.989	D	0.478
LBD	168	231				A	0.429	R	0.992	S	0.543
LBD	169	232				A	0.686	R	0.999	S	0.426
LBD	170	233				S	0.345	G	0.849	S	0.5
LBD	171	234				G	0.48	G	0.601	S	0.492
LBD	172	235				D	0.434	N	0.21	D	0.49
LBD	173	236				A	0.207	V	0.995	V	0.852
LBD	174	237				L	0.668	F	1	F	0.999
LBD	175	238				S	0.955	T	0.997	S	0.988
LBD	176	239	No	Yes	No	M	0.958	A	0.985	L	0.647
LBD	177	240	No	Yes	Yes	L	0.985	L	1	L	1
LBD-H3	178	241				P	0.991	P	1	P	1
LBD-H3	179	242				H	1	H	1	H	1
LBD-H3	180	243	Yes	Yes	Yes	L	0.633	V	0.981	L	0.447
LBD-H3	181	244	Yes	No	Yes	A	0.656	T	0.996	A	0.901
LBD-H3	182	245				D	0.983	D	0.999	D	0.999
LBD-H3	183	246	Yes	Yes	Yes	L	0.986	L	1	L	0.91
LBD-H3	184	247	Yes	Yes	Yes	V	0.515	T	0.753	S	0.746
LBD-H3	185	248				T	0.611	T	1	T	0.999
LBD-H3	186	249				Y	0.974	Y	0.984	Y	0.977
LBD-H3	187	250	Yes	No	Yes	S	0.538	M	1	M	1
LBD-H3	188	251	No	Yes	No	I	0.986	I	0.999	I	0.955
LBD-H3	189	252				Q	0.879	H	0.99	Q	0.621
LBD-H3	190	253				K	0.404	D	0.999	G	0.572
LBD-H3	191	254				V	0.886	I	0.967	V	0.882
LBD-H3	192	255				I	0.974	I	0.999	I	0.998
LBD-H3	193	256				G	0.426	A	0.985	N	0.85
LBD-H3	194	257				F	0.999	F	1	F	1
LBD-H3	195	258				A	0.953	S	0.998	A	0.981
LBD-H3	196	259				K	0.998	K	1	K	0.999
LBD-H3	197	260				M	0.969	S	0.996	V	0.373
LBD	198	261				I	0.906	L	1	I	0.569
LBD	199	262				P	1	T	0.996	P	0.941
LBD	200	263				G	0.9	D	0.579	Y	0.679
LBD	201	264				F	1	F	1	F	1
LBD	202	265				R	0.712	K	0.975	R	0.987
LBD	203	266				D	0.547	S	0.985	S	0.465
LBD	204	267				L	0.999	L	0.999	L	1
LBD	205	268				T	0.552	L	0.988	P	0.944
LBD-H4	206	269				I	0.419	I	0.999	I	0.994
LBD-H4	207	270				E	0.995	G	0.992	E	0.988
LBD-H4	208	271				D	0.997	D	0.999	D	0.999
LBD-H4	209	272				Q	1	Q	1	Q	1
LBD-H4	210	273				I	0.993	I	0.901	I	0.998
LBD-H4	211	274				A	0.637	A	0.978	S	0.973
LBD-H4	212	275				L	0.999	L	1	L	1
LBD-H4	213	276				L	0.999	L	1	L	1
LBD-H4	214	277				K	0.998	K	1	K	0.999
LBD-H4	215	278				G	0.491	G	1	G	1
LBD	216	279				S	0.944	A	0.999	A	0.985
LBD-H5	217	280				A	0.739	T	0.997	T	0.924
LBD-H5	218	281	Yes	Yes	Yes	I	0.798	F	1	F	0.881
LBD-H5	219	282				E	0.997	E	0.999	E	0.997
LBD-H5	220	283				I	0.757	V	0.974	I	0.826

Domain (DBD=DNA-binding domain LBD=Ligand-binding domain H=helix)	Site #	Human PXR amino acid #	VDR Ligand-binding residue?	PXR Ligand-binding residue?	CAR Ligand-binding residue?	AncR1 Prior to VDR/PXR split	Posterior probability (PP)	AncR2 Prior to mammalian/fish PXR split	Posterior probability (PP)	AncR3 Prior to mammalian PXR/ CXR-CAR split	Posterior probability (PP)
LBD-H5	221	284	No	Yes	Yes	I	0.66	M	1	C	0.999
LBD-H5	222	285	Yes	Yes	Yes	M	0.788	E	0.985	Q	0.902
LBD-H5	223	286				L	0.658	I	0.999	I	0.963
LBD-H5	224	287	Yes	No	No	R	0.993	R	1	R	0.974
LBD	225	288	Yes	Yes	Yes	S	0.671	F	1	F	1
LBD	226	289	Yes	No	No	N	0.993	N	1	N	1
LBD	227	290				Q	0.491	M	0.999	T	0.994
LBD	228	291	Yes	No	No	S	0.462	V	0.998	V	0.925
LBD	229	292				F	0.962	F	1	F	1
LBD	230	293				N	0.767	N	0.999	N	0.999
LBD	231	294				M	0.444	T	0.998	A	0.573
LBD	232	295				E	0.994	K	0.992	E	0.996
LBD	233	296				T	0.598	T	1	T	0.999
LBD	234					D	0.488	D	0.237	D	0.333
LBD	235	297	No	No	Yes	N	0.957	G	0.996	N	0.755
LBD	236	298				S	0.671	V	0.556	T	0.761
LBD	237	299	Yes	Yes	Yes	W	0.999	W	1	W	1
LBD	238	300				T	0.819	E	0.999	E	0.997
LBD	239	301	Yes	No	Yes	C	0.996	C	1	C	1
LBD	240	302				G	1	G	1	G	1
LBD	241	303				S	0.716	H	0.996	P	0.909
LBD	242					P	0.385	P	0.212	P	0.284
LBD	243					D	0.744	D	0.335	D	0.49
LBD	244	304				F	0.661	A	0.982	L	0.96
LBD	245	305				K	0.51	T	0.993	S	0.529
LBD	246	306	Yes	Yes	Yes	Y	1	Y	1	Y	0.998
LBD	247					Q	0.361	Q	0.168	Q	0.243
LBD	248					I	0.653	I	0.26	I	0.397
LBD	249					G	0.373	G	0.232	G	0.293
LBD	250	307	No	No	Yes	D	0.514	C	1	C	0.887
LBD	251	308	No	Yes	Yes	I	0.408	I	0.998	I	0.812
LBD	252	309				E	0.685	E	0.993	E	0.977
LBD	253	310	No	No	Yes	D	0.921	D	0.999	D	0.999
LBD	254	311	Yes	No	Yes	V	0.681	A	0.999	A	0.897
LBD	255					T	0.581	V	0.985	S	0.412
LBD	256	312				Q	0.882	R	1	R	0.319
LBD	257	313	Yes	No	No	A	0.974	A	1	A	0.971
LBD	258	314				G	0.999	G	1	G	1
LBD	259	315	Yes	No	Yes	Y	0.482	F	1	F	1
LBD	260					A	0.559	A	0.311	A	0.591
LBD	261					C	0.373	V	0.179	V	0.319
LBD	262					G	0.883	G	0.603	G	0.892
LBD	263					L	0.281	F	0.298	F	0.459
LBD	264	316	Yes	No	No	K	0.258	Q	1	Q	0.998
LBD-H7	265	317				L	0.509	P	1	Q	0.996
LBD-H7	266	318	No	No	Yes	E	0.873	L	0.998	L	0.974
LBD-H7	267	319	Yes	No	Yes	L	0.774	L	0.897	L	0.689
LBD-H7	268	320	Yes	No	No	L	0.971	L	1	L	1
LBD-H7	269	321				E	0.93	E	0.986	E	0.988
LBD-H7	270	322				P	0.997	P	1	P	1
LBD-H7	271	323	No	Yes	Yes	L	0.994	L	1	L	0.978
LBD-H7	272	324	No	Yes	Yes	L	0.838	L	0.999	L	0.999
LBD-H7	273	325				K	0.967	K	0.88	K	0.979
LBD-H7	274	326				F	1	F	1	F	1
LBD-H7	275	327	No	No	Yes	H	0.853	H	1	H	1
LBD-H7	276	328				R	0.516	H	1	Y	0.483

Domain (DBD=DNA-binding domain LBD=Ligand-binding domain H=helix)	Site #	Human PXR amino acid #	VDR Ligand-binding residue?	PXR Ligand-binding residue?	CAR Ligand-binding residue?	AncR1 Prior to VDR/PXR split	Posterior probability (PP)	AncR2 Prior to mammalian/fish PXR split	Posterior probability (PP)	AncR3 Prior to mammalian PXR/ CXR-CAR split	Posterior probability (PP)
**LBD-H7**	**277**	**329**				N	0.517	T	1	T	0.752
**LBD-H7**	**278**	**330**				M	0.748	L	1	L	0.987
**LBD-H7**	**279**	**331**				R	0.491	R	1	R	0.585
**LBD-H7**	**280**	**332**				K	0.98	N	0.99	K	0.996
**LBD-H7**	**281**	**333**				L	0.999	L	1	L	1
**LBD**	**282**	**334**				N	0.682	G	1	R	0.434
**LBD**	**283**	**335**				L	0.998	L	1	L	1
**LBD**	**284**	**336**				H	0.986	E	0.988	H	0.999
**LBD-H8**	**285**	**337**				E	0.831	E	0.999	E	0.969
**LBD-H8**	**286**	**338**				E	0.801	E	0.999	E	0.983
**LBD-H8**	**287**	**339**				E	0.997	E	0.999	E	0.999
**LBD-H8**	**288**	**340**				Y	0.824	Y	1	Y	1
**LBD-H8**	**289**	**341**				V	0.914	V	0.998	V	0.995
**LBD-H8**	**290**	**342**				L	0.998	L	1	L	1
**LBD-H8**	**291**	**343**				L	0.899	M	1	M	0.999
**LBD-H8**	**292**	**344**				M	0.61	Q	1	Q	0.763
**LBD-H8**	**293**	**345**				A	0.997	A	1	A	0.999
**LBD-H8**	**294**	**346**				I	0.882	L	0.648	M	0.864
**LBD-H8**	**295**	**347**				S	0.416	S	0.999	S	0.832
**LBD-H8**	**296**	**348**				L	0.988	L	1	L	1
**LBD**	**297**	**349**				F	0.997	F	1	F	1
**LBD**	**298**	**350**				S	0.991	S	0.999	S	0.998
**LBD**	**299**	**351**				P	0.996	P	1	P	1
**LBD**	**300**	**352**				D	0.996	D	0.999	D	0.999
**LBD**	**301**	**353**				R	0.998	R	1	R	0.998
**LBD**	**302**	**354**				P	0.999	P	0.959	P	0.999
**LBD**	**303**	**355**				G	0.996	G	1	G	0.999
**LBD**	**304**	**356**				V	0.945	V	0.999	V	0.997
**LBD**	**305**	**357**				Q	0.868	Q	1	T	0.536
**LBD**	**306**	**358**				D	0.958	E	0.935	Q	0.911
**LBD**	**307**	**359**				R	0.882	H	1	R	0.981
**LBD-H9**	**308**	**360**				Q	0.267	G	0.445	K	0.445
**LBD-H9**	**309**	**361**				R	0.874	V	0.998	V	0.916
**LBD-H9**	**310**	**362**				V	0.772	I	0.999	I	0.9
**LBD-H9**	**311**	**363**				E	0.937	D	0.999	D	0.976
**LBD-H9**	**312**	**364**				Q	0.853	K	0.592	Q	0.983
**LBD-H9**	**313**	**365**				L	0.535	I	0.998	L	0.977
**LBD-H9**	**314**	**366**				Q	0.999	H	0.999	Q	1
**LBD-H9**	**315**	**367**				E	0.961	E	0.999	E	0.999
**LBD-H9**	**316**	**368**				H	0.967	N	0.994	R	0.437
**LBD-H9**	**317**	**369**				L	0.905	M	0.806	L	0.441
**LBD-H9**	**318**	**370**				A	0.663	A	1	A	0.995
**LBD-H9**	**319**	**371**				E	0.496	L	1	L	0.979
**LBD-H9**	**320**	**372**				T	0.973	T	0.681	T	0.996
**LBD-H9**	**321**	**373**				L	0.999	L	1	L	1
**LBD-H9**	**322**	**374**				R	0.471	K	1	K	0.997
**LBD-H9**	**323**	**375**				A	0.909	T	0.999	A	0.753
**LBD-H9**	**324**	**376**				Y	1	R	0.994	Y	1
**LBD-H9**	**325**	**377**				I	0.975	I	0.999	I	0.998
**LBD-H9**	**326**	**378**				E	0.902	E	0.502	E	0.956
**LBD-H9**	**327**	**379**				C	0.992	S	0.715	C	0.994
**LBD-H9**	**328**	**380**				R	0.759	K	0.993	K	0.586
**LBD**	**329**	**381**				R	0.901	R	1	R	0.976
**LBD**	**330**					P	0.686	P	0.501	P	0.717
**LBD**	**331**	**382**				P	1	T	0.999	P	0.999
**LBD**	**332**	**383**				P	0.404	G	1	R	0.345

Domain (DBD=DNA-binding domain LBD=Ligand-binding domain H=helix)	Site #	Human PXR amino acid #	VDR Ligand-binding residue?	PXR Ligand-binding residue?	CAR Ligand-binding residue?	AncR1 Prior to VDR/PXR split	Posterior probability (PP)	AncR2 Prior to mammalian/fish PXR split	Posterior probability (PP)	AncR3 Prior to mammalian PXR/ CXR-CAR split	Posterior probability (PP)
**LBD**	**333**	**384**				P	0.964	P	1	P	1
**LBD**	**334**	**385**				E	0.465	E	0.999	E	0.804
**LBD**	**335**	**386**				N	0.442	K	1	N	0.584
**LBD**	**336**	**387**				R	0.983	H	0.948	R	0.997
**LBD**	**337**	**388**				L	0.936	M	0.992	F	0.921
**LBD-H10**	**338**	**389**				L	0.997	L	1	L	1
**LBD-H10**	**339**	**390**				Y	0.983	Y	1	Y	0.975
**LBD-H10**	**340**	**391**				A	0.742	P	1	P	0.765
**LBD-H10**	**341**	**392**				K	0.978	K	1	K	0.999
**LBD-H10**	**342**	**393**				I	0.559	V	0.958	I	0.953
**LBD-H10**	**343**	**394**				M	0.931	L	0.982	M	0.969
**LBD-H10**	**344**	**395**				E	0.593	A	0.895	A	0.778
**LBD-H10**	**345**	**396**				C	0.372	C	1	L	0.419
**LBD-H10**	**346**	**397**				L	0.999	L	1	L	1
**LBD-H10**	**347**	**398**				T	0.913	T	1	T	0.997
**LBD-H10**	**348**	**399**				E	0.937	E	0.999	E	0.999
**LBD-H10**	**349**	**400**				L	0.999	M	0.997	L	0.995
**LBD-H10**	**350**	**401**				R	0.998	R	1	R	0.997
**LBD-H10**	**351**	**402**				T	0.575	T	0.987	T	0.616
**LBD-H10**	**352**	**403**				L	0.935	M	0.955	L	0.359
**LBD-H10**	**353**	**404**				N	0.998	N	0.878	N	0.998
**LBD-H10**	**354**	**405**				E	0.703	E	0.935	A	0.422
**LBD-H10**	**355**	**406**				E	0.931	E	0.999	E	0.965
**LBD-H10**	**356**	**407**	**Yes**	**Yes**	**Yes**	**H**	**0.988**	**Y**	**0.993**	**H**	**0.95**
**LBD-H10**	**357**	**408**				S	0.856	S	0.998	T	0.664
**LBD-H10**	**358**	**409**				K	0.985	K	1	K	0.899
**LBD-H10**	**359**	**410**	**No**	**Yes**	**Yes**	**Q**	**0.99**	**Q**	**1**	**Q**	**0.989**
**LBD-H10**	**360**	**411**	**No**	**Yes**	**Yes**	**F**	**0.639**	**I**	**0.74**	**L**	**0.546**
**LBD-H10**	**361**	**412**	**No**	**Yes**	**No**	**L**	**0.973**	**L**	**1**	**L**	**1**
**LBD-H10**	**362**	**413**				Q	0.981	Q	1	Q	0.91
**LBD-H10**	**363**	**414**	**No**	**Yes**	**Yes**	**I**	**0.881**	**I**	**0.999**	**I**	**0.997**
**LBD-H10**	**364**	**415**				Q	0.294	Q	1	Q	0.998
**LBD-H10**	**365**	**416**				Q	0.33	D	0.999	D	0.994
**LBD**	**366**	**417**	**No**	**No**	**Yes**	**Q**	**0.838**	**I**	**0.998**	**I**	**0.912**
**LBD**	**367**	**418**				Q	0.671	Q	1	Q	0.631
**LBD**	**368**	**419**				E	0.303	P	1	P	0.987
**LBD**	**369**	**420**	**No**	**Yes**	**No**	**D**	**0.511**	**N**	**0.984**	**M**	**0.388**
**LBD**	**370**	**421**	**No**	**No**	**Yes**	**A**	**0.5**	**V**	**0.993**	**A**	**0.789**
**LBD**	**371**	**422**	**Yes**	**No**	**Yes**	T	0.569	I	0.687	T	0.992
**LBD**	**372**	**423**	**No**	**Yes**	**No**	P	0.915	P	0.996	P	1
**LBD**	**373**	**424**	**Yes**	**No**	**Yes**	**L**	**0.999**	**P**	**0.602**	**L**	**1**
**LBD**	**374**	**425**	**No**	**Yes**	**No**	**P**	**0.352**	**L**	**0.805**	**M**	**0.605**
**LBD**	**375**	**426**				P	0.643	L	0.855	Q	0.377
**LBD**	**376**	**427**				E	0.474	M	0.757	E	0.997
**LBD**	**377**	**428**	**Yes**	**No**	**No**	**V**	**0.712**	**E**	**0.532**	**I**	**0.47**
**LBD**	**378**	**429**				L	0.845	M	0.974	L	0.464
**LBD**	**379**	**430**				E	0.779	V	0.595	S	0.894
**LBD**	**380**	**431**				V	0.642	S	0.652	T	0.393
**LBD**	**381**	**432**	**Yes**	**No**	**No**	**F**	**0.335**	**I**	**0.393**	**M**	**0.283**
**LBD**	**382**	**433**				D	0.486	R	0.257	D	0.511
**LBD**	**383**	**434**				N	0.93	K	0.483	N	0.396
**LBD**	**384**					E	0.265	N	0.397	N	0.2
**LBD**	**385**					I	0.529	I	0.573	I	0.553
**LBD**	**386**					S	0.676	S	0.562	S	0.603
						**Average PP**	0.764	**Average PP**	0.884	**Average PP**	0.807
