# Supplementary material for: Evolution of pharmacologic specificity in the pregnane X receptor
Source: BMC Evol Biol. 2008 Apr 2;8:103. doi: 10.1186/1471-2148-8-103 (PMC2358886; doi:10.1186/1471-2148-8-103)
Supplement: Additional file 8 — Comparison of ligand-binding residues between extant and reconstructed sequences. Conservation of ligand-binding residues in extant and reconstructed PXR sequences. [file 1471-2148-8-103-S8.pdf]

Additional file 8: Conservation of 'ligand-binding residues' in extant and reconstructed sequences
See additional file 7 for complete reconstructed sequences. See Methods for additional details.

Domain	Site #	HumPXR aa #	VDR Ligand-binding residue?	PXR Ligand-binding residue?	CAR Ligand-binding residue?	Human PXR	Mouse PXR	Rat PXR	Rabbit PXR	Chicken PXR	X. laevis PXRα	X. laevis PXRβ	Zebrafish PXR	Ciona VDR/PXR	Human VDR	Lamprey VDR	AncR1	PP	AncR2	PP	AncR3	PP
LBD	91	162	Yes	No	No	F	F	F	F	F	F	F	F	Y	Y	R	Y	0.688	F	1	F	1
LBD	98	169	Yes	No	Yes	F	F	F	F	F	S	F	F	Y	F	P	F	0.987	F	1	F	1
LBD	106	177	Yes	No	No	V	V	V	V	R	R	R	R	D	R	K	R	0.969	S	0.509	R	0.98
LBD	137	206	No	Yes	No	L	S	R	L	L	-	-	W	T	D	P	S	0.605	S	0.842	L	0.915
LBD	139	208	No	Yes	No	S	P	P	T	C	-	-	F	D	C	S	S	0.514	A	0.758	S	0.764
LBD	140	209	No	Yes	No	L	M	M	M	L	-	-	N	S	I	S	S	0.527	V	0.987	M	0.433
LBD	142	211	No	Yes	No	V	I	I	L	E	-	-	T	K	S	S	T	0.307	P	0.328	T	0.432
LBD	176	239	No	Yes	No	L	L	L	L	I	M	M	S	L	M	H	M	0.958	A	0.985	L	0.647
LBD	177	240	No	Yes	Yes	L	L	L	L	L	L	L	L	F	L	V	L	0.985	L	1	L	1
LBD-H3	180	243	Yes	Yes	Yes	M	L	L	L	F	I	I	F	F	L	F	L	0.633	V	0.981	L	0.447
LBD-H3	181	244	Yes	No	Yes	A	A	A	A	A	S	S	T	C	A	T	A	0.656	T	0.996	A	0.901
LBD-H3	183	246	Yes	Yes	Yes	M	V	V	M	L	L	L	L	I	L	I	L	0.986	L	1	L	0.91
LBD-H3	184	247	Yes	Yes	Yes	S	S	S	S	S	V	F	T	M	V	M	V	0.515	T	0.753	S	0.746
LBD-H3	187	250	Yes	No	Yes	M	M	M	M	M	M	M	M	G	S	S	S	0.538	M	1	M	1
LBD-H3	188	251	No	Yes	No	F	F	F	F	I	I	L	I	I	I	I	I	0.986	I	0.999	I	0.955
LBD-H5	218	281	Yes	Yes	Yes	F	F	F	L	L	A	L	F	L	I	T	I	0.798	F	1	F	0.881
LBD-H5	221	284	No	Yes	Yes	C	C	C	C	C	S	C	I	L	I	L	I	0.66	M	1	C	0.999
LBD-H5	222	285	Yes	Yes	Yes	Q	I	I	L	Q	V	V	L	V	M	F	M	0.788	E	0.985	Q	0.902
LBD-H5	224	287	Yes	No	No	R	R	R	R	Q	R	R	H	R	R	K	R	0.993	R	1	R	0.974
LBD	225	288	Yes	Yes	Yes	F	F	F	F	F	F	F	F	S	S	A	S	0.671	F	1	F	1
LBD	226	289	Yes	No	No	N	N	N	N	N	N	N	N	Y	N	N	N	0.993	N	1	N	1
LBD	228	291	Yes	No	No	V	M	M	V	V	V	M	F	A	S	T	S	0.462	V	0.998	V	0.925
LBD	235	297	No	No	Yes	G	G	G	G	N	N	N	G	N	M	K	N	0.957	G	0.996	N	0.755
LBD	237	299	Yes	Yes	Yes	W	W	W	W	W	W	W	W	Y	W	L	W	0.999	W	1	W	1
LBD	239	301	Yes	No	Yes	C	C	C	C	C	C	C	C	S	C	L	C	0.996	C	1	C	1
LBD	246	306	Yes	Yes	Yes	Y	Y	Y	Y	F	Y	Y	Y	Y	Y	Y	Y	1	Y	1	Y	0.998
LBD	250	307	No	No	Yes	C	C	C	C	T	D	N	C	K	R	T	D	0.514	C	1	C	0.887
LBD	251	308	No	Yes	Yes	L	F	F	V	I	T	A	M	P	V	R	I	0.408	I	0.998	I	0.812
LBD	253	310	No	No	Yes	D	D	D	D	D	D	D	D	D	D	S	D	0.921	D	0.999	D	0.999
LBD	254	311	Yes	No	Yes	T	P	P	P	G	M	M	A	F	V	F	V	0.681	A	0.999	A	0.897
LBD	257	313	Yes	No	No	G	G	G	G	A	A	A	A	A	A	D	A	0.974	A	1	A	0.971
LBD	259	315	Yes	No	Yes	F	F	F	F	F	F	F	F	G	H	Y	Y	0.482	F	1	F	1
LBD	264	316	Yes	No	No	Q	Q	Q	Q	Q	R	S	Q	N	S	S	K	0.258	Q	1	Q	0.998
LBD-H7	266	318	No	No	Yes	L	L	L	L	I	L	Q	H	E	E	E	E	0.873	L	0.998	L	0.974
LBD-H7	267	319	Yes	No	Yes	L	L	L	L	Y	F	F	L	F	L	Y	L	0.774	L	0.897	L	0.689
LBD-H7	268	320	Yes	No	No	L	L	L	V	L	L	L	L	V	I	T	L	0.971	L	1	L	1
LBD-H7	271	323	No	Yes	Yes	M	L	L	L	L	L	L	M	M	L	Y	L	0.994	L	1	L	0.978
LBD-H7	272	324	No	Yes	Yes	L	M	M	L	L	V	L	M	L	I	L	L	0.838	L	0.999	L	0.999
LBD-H7	275	327	No	No	Yes	H	H	H	H	H	H	H	H	H	Q	H	H	0.853	H	1	H	1
LBD-H10	356	407	Yes	Yes	Yes	H	Q	Q	H	N	H	H	Y	F	H	C	H	0.988	Y	0.993	H	0.95
LBD-H10	359	410	No	Yes	Yes	R	Q	Q	R	Q	Q	Q	Q	N	Q	A	Q	0.99	Q	1	Q	0.989
LBD-H10	360	411	No	Yes	Yes	L	L	L	L	I	L	L	V	I	Y	F	F	0.639	I	0.74	L	0.546
LBD-H10	361	412	No	Yes	No	L	L	L	L	L	L	M	L	M	R	S	L	0.973	L	1	L	1
LBD-H10	363	414	No	Yes	Yes	I	I	I	I	I	I	I	I	L	L	V	I	0.881	I	0.999	I	0.997
LBD	366	417	No	No	Yes	I	T	S	T	L	I	I	M	N	Q	Q	Q	0.838	I	0.998	I	0.912
LBD	369	420	No	Yes	No	F	F	F	F	M	D	D	E	D	C	E	D	0.511	N	0.984	M	0.388
LBD	370	421	No	No	Yes	A	A	A	A	-	A	V	V	M	S	E	A	0.5	V	0.993	A	0.789
LBD	373	424	Yes	No	Yes	L	L	L	L	L	L	L	L	L	L	L	L	0.999	P	0.602	L	1
LBD	374	425	No	Yes	No	M	M	M	M	L	M	M	W	M	T	V	P	0.352	L	0.805	M	0.605
LBD	377	428	Yes	No	No	L	L	L	L	I	V	V	I	V	V	V	V	0.712	E	0.532	I	0.47
LBD	381	432	Yes	No	No	T	T	T	T	-	P	L	D	A	F	V	F	0.335	I	0.393	M	0.283

Abbreviations: H, helix; LBD, ligand-binding domain; PP, posterior probability
