# Supplementary material for: Evolution of pharmacologic specificity in the pregnane X receptor
Source: BMC Evol Biol. 2008 Apr 2;8:103. doi: 10.1186/1471-2148-8-103 (PMC2358886; doi:10.1186/1471-2148-8-103)
Supplement: Additional file 9 — Intrinsic disorder summary for PXRs, VDRs, and CARs. Summary data for intrinsic disorder predictions for PXR, VDR, and CAR sequences. [file 1471-2148-8-103-S9.pdf]

Additional file 9. Predicted intrinsic disorder in PXRs, VDRs, and CARs

Receptor	Species	Accession number	Disorder probability in DBD[1]	Disorder probability in LBD[1]	Disorder probability for full-length protein[1]
**PXR (NR1I2)**	**Human (*Homo sapiens*)**	Genbank:AF061056	0.826	0.259	0.447
**PXR (NR1I2)**	**Chimpanzee (*Pan troglodytes*)**	Ensembl:ENSPTRT00000028510	0.826	0.248	0.486
**PXR (NR1I2)**	**Rhesus monkey (*Macaca mulatta*)**	Genbank:AF454671	0.812	0.255	0.380
**PXR (NR1I2)**	**Mouse (*Mus musculus*)**	Genbank:AF031814	0.971	0.221	0.464
**PXR (NR1I2)**	**Rat (*Rattus norvegicus*)**	Genbank:AF151377	0.942	0.255	0.459
**PXR (NR1I2)**	**Rabbit (*Oryctolagus cuniculus*)**	Genbank:AF188476	0.942	0.197	0.406
**PXR (NR1I2)**	**Chicken (*Gallus gallus*)**	Genbank:AF276753	0.676	0.243	0.417
**PXR (NR1I2)**	**African clawed frog (*Xenopus laevis*) – BXRα**	Genbank:BC041187	0.971	**0.135**	0.433
**PXR (NR1I2)**	**African clawed frog (*Xenopus laevis*) – BXRβ**	Genbank:AF305201	0.971	0.202	0.477
**PXR (NR1I2)**	**Western clawed frog (*Xenopus tropicalis*)**	Ensembl: ENSXETT00000039109	1.000	0.333	0.564
**PXR (NR1I2)**	**Medaka (*Oryzias latipes*)**	Ensembl:ENSORLT00000022473	0.912	0.414	0.547
**PXR (NR1I2)**	**Zebrafish (*Danio rerio*)**	Genbank:NM_001098617	1.000	0.253	0.493
**VDR/PXR (NR1I1/2)**	**Sea squirt (*Ciona intestinalis*)**	Genbank:AB210742	1.000	0.270	0.421
		**Average (excluding *Ciona* VDR/PXR)**	0.904	0.251	0.464
		**Standard deviation**	0.099	0.070	0.054
**VDR (NR1I1)**	**Human (Homo sapiens)**	Genbank:NM_000376	0.926	0.432	0.557
**VDR (NR1I1)**	**Rhesus monkey (macaca mulatta)**	Ensembl:ENSMMUT00000009414	0.926	0.432	0.569
**VDR (NR1I1)**	**Tamarin monkey (Saquinus oedipus)**	Genbank:AF354232	0.926	0.429	0.555
**VDR (NR1I1)**	**Guinea pig (Cavia porcellus)**	Ensembl:ENSCPOT00000006089	0.941	0.439	0.569
**VDR (NR1I1)**	**Mouse (Mus musculus)**	Genbank:NM_009504	0.926	0.407	0.540
**VDR (NR1I1)**	**Rat (Rattus norvegicus)**	Genbank:NM_017058	0.912	0.422	0.548
**VDR (NR1I1)**	**Chicken (Gallus gallus)**	Genbank:AF011356	0.882	0.486	0.592
**VDR (NR1I1)**	**Japanese quail (Corturnix japonica)**	Genbank:U12641	0.985	0.460	0.614
**VDR (NR1I1)**	**African clawed frog (Xenopus laevis)**	Genbank:U91849	0.956	0.438	0.562
**VDR (NR1I1)**	**Western clawed frog (Xenopus tropicalis)**	Ensembl:ENSXETT00000023342	1.000	0.340	0.521
**VDR (NR1I1)**	**Bastard halibut (Paralichthys olivaceus)**	Genbank:AB037674	0.985	0.498	0.624
**VDR (NR1I1)**	**Carp (Cyprinus carpio)**	Genbank:AJ784084	0.897	0.414	0.552
**VDR (NR1I1)**	**Salmon (Salmo salar)**	Genbank:AJ780914	0.882	0.490	0.567
**VDR (NR1I1)**	**Stickleback fish (Gastrosteus aculeatus)**	Ensembl:ENSGACT00000006308	0.941	0.502	0.612

**VDR (NR1I1)**	**Zebrafish (Danio rerio)**	Genbank:AF164512	0.882	0.447	0.574
**VDR (NR1I1)**	**Sea lamprey (Petromyzon marinus)**	Genbank:AY249863	0.912	0.434	0.567
**VDR/PXR (NR1I1/2)**	**Sea squirt (Ciona intestinalis)**	Genbank:BR000137	1.000	0.270	0.421
		**VDR Average (excluding Ciona VDR/PXR)**	0.930	0.442	0.570
		**VDR Standard deviation**	0.037	0.040	0.028
**CAR (NR1I3)**	**Human (Homo sapiens)**	Genbank:NM_005122	0.647	0.240	0.374
**CAR (NR1I3)**	**Chimpanzee (Pan troglodytes)**	Ensembl:ENSPTRT00000002884	0.647	0.237	0.374
**CAR (NR1I3)**	**Rhesus monkey (macaca mulatta)**	Genbank:AY116212	0.529	0.185	0.330
**CAR (NR1I3)**	**Cow (Bos taurus)**	Ensembl:ENSBTAP00000012145	0.721	0.189	0.361
**CAR (NR1I3)**	**Dog (Canis familiaris)**	Ensembl:ENSCAFT00000020528	0.779	0.189	0.398
**CAR (NR1I3)**	**Pig (Sus scrofa)**	Genbank:AB214979	0.691	0.188	0.353
**CAR (NR1I3)**	**Mouse (Mus musculus)**	Genbank:NM_009803	0.588	0.204	0.358
**CAR (NR1I3)**	**Rat (Rattus norvegicus)**	Genbank:NM_022941	0.603	0.224	0.377
**CAR (NR1I3)**	**Rabbit (Oryctolagus cuniculus)**	Ensembl:ENSOCUT00000013535	0.706	0.177	0.347
**CAR (NR1I3)**	**Squirrel (Spermophilus tricemlineatus)**	Ensembl:ENSSTOT00000003812	0.647	0.265	0.395
**CAR (NR1I3)**	**Hedgehog (Erinaceus europaeus)**	Ensembl:ENSEEUT00000004981	0.705	0.220	0.401
**CAR (NR1I3)**	**Baikal seal (Phoca sibirica)**	Genbank:AB109553	0.794	0.155	0.356
**CAR (NR1I3)**	**Northern fur seal (Callorhinus ursinus)**	Genbank:AB109554	0.794	0.147	0.351
		**CAR Average**	0.681	0.202	0.367
		**CAR Standard deviation**	0.081	0.034	0.022

[1] Proportion of amino acid residues in DNA-binding domain (DBD), ligand-binding domain (LBD), or full-length protein that have disorder probability greater than 0.50.

Disorder calculations were calculated as previously described [1, 2]. Ensembl sequences can be found at http://www.ensembl.org.

References

1. Peng K, Vucetic S, Radivojac P, Brown CJ, Dunker AK, Obradovic Z: **Optimizing long intrinsic disorder predictors with protein evolutionary information**. *J Bioinform Comput Biol*, 2005, **3:**35-60.
2. Dunker AK, Cortese MS, Romero P, Iakoucheva LM, Uversky VN: **Flexible nets: the roles of intrinsic disorder in protein interaction networks**. *FEBS Lett*, 2005, **270:**5129-5148.
